# Analysis of CTCL cell lines reveals important differences between mycosis fungoides/Sézary syndrome *vs. HTLV-1^+^* leukemic cell lines

**DOI:** 10.18632/oncotarget.21619

**Published:** 2017-10-07

**Authors:** Elena Netchiporouk, Jennifer Gantchev, Matthew Tsang, Philippe Thibault, Andrew K. Watters, John-Douglas Matthew Hughes, Feras M. Ghazawi, Anders Woetmann, Niels Ødum, Denis Sasseville, Ivan V. Litvinov

**Affiliations:** ^1^ Division of Dermatology, McGill University, Montréal, Québec, Canada; ^2^ Division of Dermatology, University of Ottawa, Ottawa, Ontario, Canada; ^3^ Université de Sherbrooke Rnomics Platform, Sherbrooke, Québec, Canada; ^4^ Department of Pathology, McGill University Health Centre, Montreal, Québec, Canada; ^5^ Department of International Health, Immunology, and Microbiology, University of Copenhagen, Copenhagen, Denmark

**Keywords:** human T-cell lymphotropic virus type 1, cutaneous T-cell lymphomas, spectral karyotyping, gene expression analysis, xenograft tumors

## Abstract

*HTLV-1* is estimated to affect ~20 million people worldwide and in ~5% of carriers it produces Adult T-Cell Leukemia/Lymphoma (ATLL), which can often masquerade and present with classic erythematous pruritic patches and plaques that are typically seen in Mycosis Fungoides (MF) and Sézary Syndrome (SS), the most recognized variants of Cutaneous T-Cell Lymphomas (CTCL). For many years the role of *HTLV-1* in the pathogenesis of MF/SS has been hotly debated. In this study we analyzed CTCL vs. *HTLV-1^+^* leukemic cells. We performed G-banding/spectral karyotyping, extensive gene expression analysis, TP53 sequencing in the 11 patient-derived *HTLV-1^+^* (MJ and Hut102) vs. *HTLV-1^-^* (Myla, Mac2a, PB2B, HH, H9, Hut78, SZ4, Sez4 and SeAx) CTCL cell lines. We further tested drug sensitivities to commonly used CTCL therapies and studied the ability of these cells to produce subcutaneous xenograft tumors in NOD.Cg-Prkdc^scid^ Il2rg^tm1Wjl^/SzJ mice. Our work demonstrates that unlike classic advanced MF/SS cells that acquire many ongoing balanced and unbalanced chromosomal translocations, *HTLV-1*^+^ CTCL leukemia cells are diploid and exhibit only a minimal number of non-specific chromosomal alterations. Our results indicate that *HTLV-1* virus is likely not involved in the pathogenesis of classic MF/SS since it drives a very different pathway of lymphomagenesis based on our findings in these cells. This study also provides for the first time a comprehensive characterization of the CTCL cells with respect to gene expression profiling, TP53 mutation status, ability to produce tumors in mice and response to commonly used therapies.

## INTRODUCTION

Viruses are known to cause a number of human cancers, where Human Papilloma Virus (HPV), Human Herpesvirus 8 (HHV8) *and* Merkel Cell Polyomavirus *were shown to cause Squamous Cell, Kaposi Sarcomas and Merkel Cell cancers, respectively. Similarly,* *Epstein-Barr Virus (EBV)* and *Human T-Cell Lymphotropic Virus Type 1 (HTLV-1)* were implicated as causes for a number of hematologic malignancies [1–3], where specifically *HTLV-1* is known to cause Adult T-Cell Leukemia/Lymphoma (ATLL), a WHO recognized variant of Cutaneous T-Cell Lymphoma (CTCL)[4, 5]. It is estimated that ~20 million people are infected with *HTLV-1* around the world. This virus is prevalent in Central and West Africa, in the Caribbean, in Central and South America, in native populations in Canada, and among intravenous drug users in the United States [6].

Cutaneous T-Cell Lymphoma (CTCL) is the most common lymphoma of the skin [7]. Most CTCL variants (with a notable exception of ATLL and Extranodal NK/T-cell lymphoma, nasal type) are not caused by an *HTLV-1* or another virus. CTCL was reported to be more common in HIV^+^ individuals [7–9]. Mycosis Fungoides (MF), its leukemic form, Sézary Syndrome (SS), and primary cutaneous anaplastic large cell lymphoma (cALCL) are the most common forms and account for ~80% of all CTCL [10].

MF typically presents with erythematous patches and plaques on the trunk following a bathing suit distribution, but as the disease progresses skin involvement can become confluent and patients can develop erythrodermic disease. MF can involve skin, lymph nodes, blood, bone marrow and visceral organs. SS is characterized by a triad of erythroderma, lymphadenopathy and detection of malignant T cells with cerebriform nuclei on a peripheral blood smear [5].

Before, SS was viewed as being on the same continuum with MF and was mostly regarded as an aggressive leukemic form of MF. However, as highlighted recently, SS is now considered separate from MF and erythrodermic/leukemic MF [11]. SS typically arises de novo and evolves in a short time period, although some patients may have a prodrome of pruritus, erythema and nonspecific dermatitis [11, 12]. It also has a much worse overall prognosis than the leukemic MF. In Caucasians MF/SS primarily affect individuals over 55 years of age, while in African-Americans, Hispanics and Arabic individuals this disease presents at a significantly younger age [13–15].

*HTLV-1* in ~5% of carriers produces ATLL, which can have variable presentations and can often masquerade and present with classic MF-like erythematous pruritic patches and plaques. This can lead to misdiagnosis of Mycosis Fungoides. In fact, in two high profile reports published in *Science* and *PNAS* journals, two patients (a 50 year-old Caucasian male form Boston, MA and a 28 year-old African-American male presenting to the National Cancer Institute Veterans Administration hospital in Bethesda, MD) were diagnosed with “advanced Mycosis Fungoides” and have provided critical samples to establish MJ and Hut102 CTCL cell lines, respectively. These cell lines were later found to harbor *HTLV-1* virus and, hence likely represent ATLL, not Mycosis Fungoides. Despite that, on the American Tissue Culture Collection (ATCC) website MJ is listed as “Cutaneous T Cell Lymphoma; Mycosis Fungoides” cell line and Hut102 is listed as “Lymphoma; Mycosis Fungoides” and not ATLL cell line. *HTLV-1* serologic screening plays a key role to establish the diagnosis of ATLL in patients and without this test distinguishing the two lymphomas in North America would be very challenging in the clinic. To further complicate these matters, MF can exhibit an identical clinical course as the smoldering, chronic, lymphoma and leukemic variants of ATLL.

Distinguishing these lymphomas and better understanding their biologic differences is not of mere academic interest since ATLL poses a much greater risk to patients than MF, and may require earlier initiation of multimodality chemotherapy treatments and/or bone marrow stem cell transplantation. In addition, even in healthy *HTLV-1* carriers impaired immune responses against *EBV* have been observed [16]. One way the virus leads to immunodeficiency is by infecting CD8^+^ T-lymphocytes, which impairs their function [17]. At last, it is important to distinguish these cancers to appropriately council our patients about precautions to decrease the risk of *HTLV-1* transmission. Hence, it is critical to better understand the fundamental differences between *HTLV-1^+^* *vs.* *HTLV-1^-^* lymphomas/leukemias. The current study was designed to highlight key molecular differences between classic MF/SS disease and *HTLV-1*^+^ leukemias.

## RESULTS

Patient-derived cells are indispensable and are routinely used as robust and reproducible models to study human cancers. For CTCL, in particular, 11 patient-derived cell lines are available to study this malignancy [18–20]. As discussed in the introduction, two of the cell lines (MJ and Hut102) harbor *HTLV-1* virus. Furthermore, these cell lines remain poorly characterized and it remains not well elucidated which cell lines represent Mycosis Fungoides *vs.* Sézary Syndrome variants of CTCL. First and foremost, we have reviewed the original publications describing these cell lines. A summary of data from these papers is presented in Supplementary Table 1 and in Supplementary Materials of this report. Based on the available description of these patients, it appears that MyLa cells represent advanced extensive Mycosis Fungoides skin disease, Mac2A and PB2B lines represent advanced CD30^+^ lymphoproliferative skin lymphoma, HH cells represent aggressive leukemic MF, while SZ4/Sez4, Hut78/H9 and SeAx cells represent Sézary Syndrome. Notably, as discussed above, Hut102 and MJ cells were indicated to represent Mycosis Fungoides, but were found to have *HTLV-1* virus.

### Use of RT-PCR assay to detect HTLV-1 virus in patient-derived cell lines

First and foremost, we used the previously described RT-PCR assay that is based on the detection of expression of *Tax*, *Gag*, *Pol*, *Env* and *pX* genes to identify which cells harbor *HTLV-1* virus [21, 22]. This analysis demonstrated that out of 11 tested cell lines only MJ and Hut102 cells expressed these genes and are infected by *HTLV-1* (Supplementary Figure 1). Importantly, this assay confirmed that Myla, Mac2a, PB2B, HH, H9, Hut78, SZ4, Sez4 and SeAx cells are *HTLV-1* negative.

### Structural karyotype analysis of the 11 patient-derived cell lines

A number of important chromosomal alterations were reported in MF and SS patients with a number of important differences documented between MF and SS [23–28]. Hence, we wanted to perform G-band analysis and spectral karyotyping in our panel of CTCL cell lines. For each cell line we analyzed five cells by spectral karyotyping. This analysis revealed that all CTCL cell lines except the *HTLV-1^+^* MJ and Hut102 cells demonstrate significant and ongoing genomic instability, where multiple different chromosomal changes are observed between these five cells within the same cell line. Summary of all karyotype data is presented in Table 1, while detailed karyotype findings are presented in Supplementary Tables 2-3.

**Table 1:** Results of G band and spectral karyotyping analyses for the 11 patient-derived CTCL cell lines

Cell line	G to Sky karyotype findings
MyLa	46~48,XY,der(1)(1pter→1q32:)[5],der(1)t(1;15)(p36.2~36.3;q15)[5],der(2)t(2;14)(q32~33;q12~13)[5],t(4;5)(q13;q14)[5], der(5)t(5;?)(q35;?)[5], del(6)(q15q23)[5], del(7)(p15)[5],del(9)(p21)[5],der(10)t(10;13)(p14;q13)[5], der(13)t(13;14)(q33;q21)[5],der(13)t(13;16)(q14;?q23)[5], der(14)(2pter→2p13::14p12→14q12~13::2q32~33→2qter)[5], der(14)t(14;18)(q22;q22)[4],der(15)t(1;15)(q32;q15)[5],+17 [5], der(18)t(18;?19)(q23;p13.1~13.2)[5],der(19)t(?11;19)(?;p13.1~13.2)[5], der(20)t(20;?)(q13.3;?)[5], der(21)t(20;21)(?p11.2;q22.3)[5],?psu dic(16;22)(p10;p13)[5][cp5]
Mac2A	43~44,X,-Y [5],t(3;7)(q22~23;q21.2~22)[5],del(6)(q15)[5], der(6)t(6;22)(p21.1~21.2;q11.1)[5], der(8)(?9?→?9?::8p22→8qter)[5], der(9)(8pter→8p22::9p24→9q34::?5?→?5?)[4],der(10)(?acro-p::10q10→10qter)[5],der(10)(10pter→10q2?6::10q2?6→10q2?1::12q21→12qter)[5], der(11)t(11;14)(q22;q13~21)[5], del(12)(q11q13)[5],-14 [5], der(15)t(2;15)(p11.2;p12)[5], del(16)(q11.2q22)[5], ?dup(16)(q21q24)[5], der(20)(:20?q13.1→20?q13.1::20p13→20q?13.1::20q?13.1→20qter)[5], -22 [5],der(22)(6?pter→6?p21.3::22q10→22q11.2::22q13.1→22qter)[5], +der(?)(?acro-p→?cen::?cen→?acro-p)[4][cp5]
PB2B	45,X,-Y [5],t(1;17)(p34.3~35;q21)[4],t(2;10)(p24;q25.3~26.1)[5], der(3)t(1;3)(q12~21;q24)[2] or der(3)(3pter→3q24:)[3], +der(5)(:5q10→5qter)[5], der(5;6)(5pter→5p10::6q10→6q15:)[5],inv(7)(p11.2p22)[5],der(8)(?9?→?9?::8p22→8qter)[5], der(9)(8pter→8p22::9p24→9q22::3q23→3qter)[5], der(10)t(6;10)(?;q10)[5],del(11)(p15.2~15.4)[4], der(12)(12pter→12q11::12q13→12qter)[5], der(12)(12pter→12q13::12q13→12q11::12q13→12qter)[4], der(15)t(2;15)(p11.2;p12)[5],del(16)(q11.2q22)[5], der(18)t(6;18)(p12;p11.3)[5],der(18)t(12;18)(?;q22)[4], der(20)(:20?q13.1→20?q13.1::20p13→20q?13.1::20q?13.1→20qter)[5], der(21)t(9;21)(q22;p12)[5],der(22)t(14;22)(q22;q10)[5][cp5]
HH	44~45,X,-Y [5],?del(2)(q3?2q3?3)[5], der(3)t(2;3)(p?14~21;p25~26)[5], der(3)(3pter→3p10::12q21→12qter)[4], der(4) (4pter→4q21::4q?→4q?::4q21→4q31::4q?→4q?::4q31→4q3?3::1p35~36.1→1pter)[5], der(5)(10qter→10q24::9q34→9q13::5p15→5qter)[5],t(6;14)(q23~24;q23~24)[5],+8 [5], der(9)(9pter→9p10::?acro-p)[5], der(10)(10pter→10q24:)[5],der(12)(12pter→12q21::3q11.2→3qter)[5], der(14)(::14q13→14q10::14q13→14qter)[5],-15 [5], der(17)t(?11;17)(q23;q25)[5],der(18)t(3;18)(q24;q22)[5][cp5]
SZ4	79~81<4n>,X or XX,+der(X)(Xpter→Xq23~24:)[2] or der(X)(Xpter→Xq23~24:)[1] or der(X;20)(p10;q10)[2],-X [5],-X [5], +1 [5],der(1)(3pter→3p21::?→?::3p21→3p13~14::1p31→1q42::8p21→8pter)[5], der(1)(6qter→6q23::1p32~34→1qter)[5],psu dic(1;16)(16pter→16q24::1q22~25→1q10::1q10→1qter)[5], t(1;11;8)(q42;q23;p21)[5], der(3)t(3;6)(q12~13;?)[5], der(3;10)(q10;q10)[2],der(4)t(4;20)(q33;?q13.1)[5], ?inv(4)(q33q35)[5],psu dic(4;21)(q10;p12~13)[5], der(5)(5pter→5p10::5q35→5q31::1?→1?::7?→7?::5q35→5q31:)[4], der(6)(6pter→6p21.3::6p21.1→6q23~24::1p34→1pter)x2 [5], t(6;11)(q16~21;q21~22)[5],-7 [5],der(7)t(7;20)(q22;p11.1~11.2)[3],-8 [5],ins(8;18)(q11.2;?)[2],der(9)t(9;14)(p13~22;q13)x2 [5], der(10)(10pter→10q24:)x1 [2] or x2 [3], der(10;17)(17pter→17p10::10q10→10q24:)[2], der(11)(:14q?32→14q?24::11p15→11q21~22:)[5],-12 [5],-13 [3], der(13)(:13q?31→13q?14::13p12→13qter)[5], -14 [5],-14 [5],der(14)t(7;14)(p13~15;q10)[5], der(15)(10qter→10q24::15p12→15qter)[5], del(16)(q11.1)[3], der(17)(17qter→17q10::17q10→17q25::?3?q13.3→?3q21:)[2] or der(17)(17pter→17q25::3?q13.3→3q26::13q31→13qter)[3], i(17)(q10)[3],+der(18)(acro-p::18?→18?::7q22→7qter)[3], der(18) (8qter→8q11.2::18?p11.3→18?q12::Xq2?8→Xq2?3~2?4::3q13.2~13.3→3qter)[3], der(18)t(8;18)(q11.2;p11.3)[5], -19 [5],der(19)t(7;19)(p11;q13.4)[4], +21 [5],der(21)t(9;21)(p22;q22)x2 [5],-22 [5], +der(?)(acro-p::8?q13→8?q22:)[5][cp5]
Sez4	77~80<3n>,XX [2] or t(X;X)(p22.2;p22.3)[2],-?X [5],+psu dic(1;16)(16pter→16q24::1q22~25→1q10::1q10→1qter)[5], der(1)(3pter→3p21::?→?::3p21→3p13~14::1p31→1qter)[5], der(1)(1pter→1q42::8p21→8pter)[2] or t(1;11;8)(q42;q23;p21)[2], +2 [4],+der(3)t(3;6)(q12~13;?)[5],+der(4)t(4;7)(q23~27;q22)[2] or der(4)t(4;7)(q23~27;q22)[2],der(4)t(4;10)(p15;q25)[3],+der(5) (5pter→5p10::5q35→5q31::1?→1?::7?→7?::5q35→5q31:)[5], der(6)(6pter→6p21.3::6p21.1→6q23~24::1p34→1pter)[5], der(6)(6pter→6p21.3::6p21.1→6p10::7p13→7pter)[2], t(6;11)(q16~21;q21~22)[5], -7 [3],i(7)(q10)[2],der(8)t(8;11)(p21;q23)[2], +del(9)(q12)[5],der(9)t(9;14)(p13~22;q13)x2 [5], +10 [2] or +der(10)t(4;10)(q28;q25)[3],der(10)(10pter→10q24:)x2 [5], +der(11)(:14q?32→14q?24::11p15→11q21~22:)[3] or der(11)(:14q?32→14q?24::11p15→11q21~22:)[1], der(13)(:13q?31→13q?14::13p12→13qter)[3],-14 [5],der(15)(10qter→10q24::15p12→15qter)[5], del(16)(q11.1)[5],+i(17)(q10)[4] or i(17)(q10)[1],der(18)t(8;18)(q11.2;p11.3)x2 [5], der(19)t(7;19)(p11;q13.4)[5],+20 [5], +der(21)t(9;21)(p22;q22)x2 [5],+der(?)(acro-p::8?q13→8?q22:)[5][cp5]
SeAx	64~71<3n>,X,-X [5],der(X;16)(q10;p10)[5], +der(1)t(1;2)(p10;?)x1 [1] or x2 [3], der(1)(:1q21→1p36.1::1q21→1qter)[5],der(1)(12qter→12q21~23::1p21→1q21:)[5],i(1)(q10)[5], +der(2;19)(6qter→6q22::2?→2?::19q10→19qter)[3] or der(2;19)(6qter→6q22::2?→2?::19q10→19qter)[2],der(2)(6qter→6q22::2?→2?::16q11.2~13→16qter)[4], der(2)(11qter→11q14~21::2?→2?::2?p25→2?qter)x1 [2] or x2 [3], +der(3;17)(p10;p10)[4] or der(3;17)(p10;p10)[1], der(3)t(X;3)(p11.4~21;q23~24)[4], der(3)(3pter→3q12::3?→3?::?11 or 17?→?11 or 17?::3?→3?::9q13~21→9qter)[5],-4 [5],der(4)t(1;4)(p36.1;p16)[4],+der(5)t(5;8)(p13;p22~23)[4],+der(6)(6pter→6q10::6?→6?::2q22~23→2qter)[4] or der(6)(6pter→6q10::6?→6?::2q22~23→2qter)[1],der(6)(6pter→6q22:)x1 [1] or x2 [4],der(6)(6pter→6q22::2?→2?::6?→6?::16?→16?)[5], der(7)(7pter→7q32::10q22→10q24::5p13→5pter)[5],der(8)t(8;10)(p22~23;q24)x3 [5], -9 [5], der(10)t(5;10)(p13;q24)x2 [5], der(10)(10pter→10q24:)[4], der(11)t(2;11)(?;q14~21)x2 [5],der(12)t(10;12)(?;p12~13)[5], -13 [5],der(14)t(12;14)(p12~13;p12)[4],+der(17)t(15;17)(?;p10)[4] or der(17)t(15;17)(?;p10)[1],der(17)t(3;17)(q12;q10)x2 [5], -18 [5],+20 [4][cp5]
Hut78	71~75<3n>,-Y [5],t(X;13)(p11.2~11.4;q14)x1 [1] or x2 [4],+der(2;20)(p10;p10)[5], der(2)(2pter→2q21::2?q31→2?q33::8q24.1→8qter)[5],+3 [5], der(3)(3pter→3q29::10q24→10qter)x1 [1] or x2 [4], +?4 [5],?4 [5], der(4)(4pter→4p?14::4?q21→4?p14::4?q25→4?q21::16p?11.1→16p?13.3::13q14→13qter)[5], der(4) (4pter→4p?14::4?q21→4?p14::4?q25→4?q21::16p?11.1→16p?13.1::6?→6?::11p11.2→11pter)[5], -5 [3],der(5)t(5;8)(?;?)[2], t(5;6)(p10;p10)x2 [5], +der(6) (4qter→4q21~24::6?p21→6?q13::6?q23→6qter)x2 [5], +der(7)t(7;8)(q11.2;q24.1)[5],der(7)(10?→10?::7p14~15→7qter)x2 [5],der(8)(?5?→?5?::8q10→8q22~24.1::5?q33→5?qter)[2] or der(8;20)(20qter→20q10::8q10→8q22~24.1::5?q33→5?qter)[2],-9 [5], der(9)(Y?→Y?::9p21~22→9qter)x2 [5], der(10)t(7;10)(q11.2;q22.2~22.3)[5], der(10)(10pter→10q24:)[5],der(11)(?16?→?16?::11?p12~14→11p10::11q21→11qter)[3],?del(12)(p13)[2],-13 [4],+14 [5],?del(14)(q?11.2q?24)x2 [5],+15 [3], -16 [5], +17 [5],+der(18)t(18;20)(?q11.2;?p11.2)[2],der(18)t(2;18)(?p22~23;p11.2~11.3)x1 [1] or x2 [4],i(18)(p10)[4], +der(19)t(19;20)(q13.?3;q11.2)[5], der(19)t(19;20)(q13.?3;q11.2)[5],der(19;22)(q10;q10)x2 [5],+del(20)(q11.2q13.1)[4], del(20)(q11.2q13.1)[1],+der(20;21)(20pter→20p10::21q10→21q22::8?q24.1→8?qter)[4], der(20)(:9p11→9p24::20p13→20qter)x2 [5], der(20)(20pter→20q10::20?→20?::20q12→20qter)[2], der(21)(11qter→11q13~14::21q10→21qter)[4][cp5]
H9	60~70<3n>,-Y [5],der(X)t(X;13)(p11.2~11.4;q14)x2 [5],der(1)(1pter→1q31::1?→1?)[5], der(2)(2pter→2q21::2?q31→2?q33::8q24.1→8qter)x2 [5], der(3)(3pter→3q29::10q24→10qter)x1 [1] or x2 [4], ?4 [5],?4 [2],der(4)(4pter→4q22~24::?6?→?6?)[2], der(4)(4pter→4p?14::4?q21→4?p14::4?q25→4?q21::16p?11.1→16p?13.3::13q14→13qter)[5],t(5;6)(p10;p10)x1 [1] or x2 [4],der(5)(5pter→5p10::?acro-p)[2], +der(6)(4qter→4q21~24::6?p21→6?q13::6?q23→6qter)x1 [1]or x2 [4], der(6)(:9p11→9p24::6p21.3→6qter)[5], +der(7)(7pter→7p10::3q13.2→3q29::10q24→10qter)[5],der(7)(10?→10?::7p14~15→7qter)x2 [5],der(7)(7pter→7q31~32::17?→17?)[5], der(8)t(?2;8)(?;q24.1~24.2)[3],-9 [5],der(9)(Y?→Y?::9p21~22→9qter)x1 [2] or x2 [3],+der(10)(10pter→10q24:)[4] or der(10)(10pter→10q24:)[1], der(10)(6qter→6q21::10p11.2→10q24::6q21→6qter)[3], der(10)t(7;10)(q11.2;q22.2~22.3)x2 [5], ?del(11)(q?13~14)x2 [5],-13 [5],der(13)(10qter→10q24~25::2q33→2q14.3~21::13q10→13q14::Xp11.2~11.4→Xpter)[5],der(13)t(X;13)(p11.2~11.4;q14)[4], ?del(14)(q?11.2q?24)[5],-15 [5],-16 [5],+17 [4], +der(18)t(15;18)(q1?5;q10)[2],i(18)(q10)[4],t(18;18)(p10;p10)[4], +der(19)t(19;20)(q13.?3;q11.2)[5],t(19;20)(q13.?3;q11.2)[5],der(19;22)(q10;q10)x2 [5],+der(20)t(6;20)(p21.3;p13)[3] or der(20)t(6;20)(p21.3;p13)[1], der(20)(:9p11→9p24::20p13→20qter)[4], der(20)(20pter→20q10::20?→20?::20q12→20qter)[4], der(21)(11qter→11q13~14::21q10→21qter)[4][cp5]
MJ	42~47,XY,der(17)t(2;17)(p11.2;q25)[5][cp5]
Hut102	46 or 92,XY or XXYY,der(6)(6pter→6q?21::6q?27→6q?21::6q?23→6q?21:)x1 [3] or x2 [2][cp5]

As alluded to above, one striking observation was that MyLa, PB2B, Mac2A, HH, Hut78, H9, SeAx, Sez4 and SZ4 cells are mostly aneuploid and have a large number of alterations, while Hut102 and MJ *HTLV-1^+^* cells are mostly diploid and have very few structural alterations. This dramatic difference highlights that even though MJ and Hut102 cells were derived from the “so called” MF CTCL patients they are vastly different form the classic MF/SS disease as represented by the other 9 patient-derived cell lines. Indeed, previous reports did indicate a paucity of chromosomal alterations in ATLL patients [29, 30]

Next, based on this analysis we wanted to identify chromosomal abnormalities that are common for a given cell line and, more importantly, determine chromosomal alterations that are seen across multiple cell lines. These findings are presented in Supplementary Tables 2-3. These results demonstrate that, as expected, H9 and Hut78 (H9 is a clonal line derived from Hut78) are almost identical structurally based on the number of shared chromosomal aberrations. Similarly, PB2B and Mac2A have a very similar number and types of chromosomal aberrations, which highlights that they represent the same clinical disease, but at different time points, as indicated in the original report [31, 32]. Notably, this analysis further confirms that, indeed, these biopsied skin tumors in this patient were caused by the same T cell clone [31, 32]. Also, our karyotype analysis confirms that SZ4 and Sez4 are essentially the same cell line that at some point acquired different names (Supplementary Tables 2-3).

### Karyotype comparison of cell lines and human patient data

As demonstrated by the above analysis, 9 patient derived MF/SS cell lines have numerous chromosomal abnormalities and exhibit ongoing genomic instability, as highlighted by many changes that are seen sporadically in different cells within the same cell line (Table 1). In contrast, this was not seen in MJ and Hut102 *HTLV-1*^+^ cells. One question emerges is whether these structural findings reflect the clinical disease that is observed in MF/SS patients? Furthermore, it is recognized that certain structural chromosomal aberrations are more common in MF than SS. Can the presence or absence of these chromosomal aberrations help define cell lines as being representative of MF skin, lymph node *vs.* SS blood/leukemic disease?

To answer these questions we compared our karyotype cell line findings to karyotype analysis of patients that were described in 15 seminal manuscripts [23–27, 33–42]. The number of selected papers is not exhaustive, but provides a broad representation of karyotype anomalies seen in MF and SS patients. Our detailed findings are presented in Supplementary Table 4. From this analysis it is evident that, like patient-derived cell lines, MF and SS patients universally exhibit a great number of chromosomal gains, losses, balanced and unbalanced translocations and other structural aberrations. This analysis demonstrates striking disease heterogeneity, where only few chromosomal alterations were noted across multiple studies (e.g., loss of 10q24, 9p21 etc.), while most chromosomal alteration are only reported to occur sporadically [23–28].

Notably, every time an aberration was seen in MF/SS patients, aberrations in the same regions were also observed in the studied patient-derived cells. Importantly, chromosomal losses in 1p36.1, 9p21, 13q14 and 16q24 regions that were commonly seen in MF and SS patients were also present in 2-5 out of 11 studied CTCL cell lines (Supplementary Table 4). Interestingly, losses and gains that were seen in 10q24 region in SS patients were also observed in blood-derived SeAx, Sez4, SZ4, H9, Hut78 and HH cells, but not in skin-derived MF MyLa, Mac2A and PB2B cells. Hence, loss of 10q24 region could be a structural change marker indicative of a leukemic disease. Importantly, this analysis indicates that chromosomal aberrations (number of abnormalities and their nature) seen in cell lines are representative of karyotype changes that are routinely observed in MF/SS patients.

In summary, 9 MF/SS cell lines appear to represent six clinical CTLC cases: 1) MyLa, 2) Mac2A/PB2B, 3) HH, 4) Hut78/H9, 5) SeAx and 6) Sez4/SZ4, while Hut102 and MJ genetically differ greatly from classic MF/SS disease and represent ATLL.

### Gene expression changes and clustering of the MF/SS cell lines

In recent years a number of studies performed analysis of gene expression in MF/SS patients in order to identify novel diagnostic/prognostic markers and to better understand oncogenes and tumor suppressor genes involved in lymphomagenesis [18–20, 43–50]. This work highlighted many genes, whose expression is believed to be important in CTCL (e.g., TOX, GTSF1, LCK, FYB, CCR4, ITK, CD30/TNFRSF8, etc.), which are described in the supplementary tables of our previous reports [18, 20, 44, 50]. These genes were time and again validated across multiple studies to play an important role in CTCL pathogenesis. Based on our previous work, we have performed gene expression analysis of the selected 107 selected genes in the 11 patient-derived cell lines and have performed unsupervised clustering analysis based on these expression findings. MJ cells were used as a reference since they showed detectable expression for all 107 genes.

**Figure 1:**
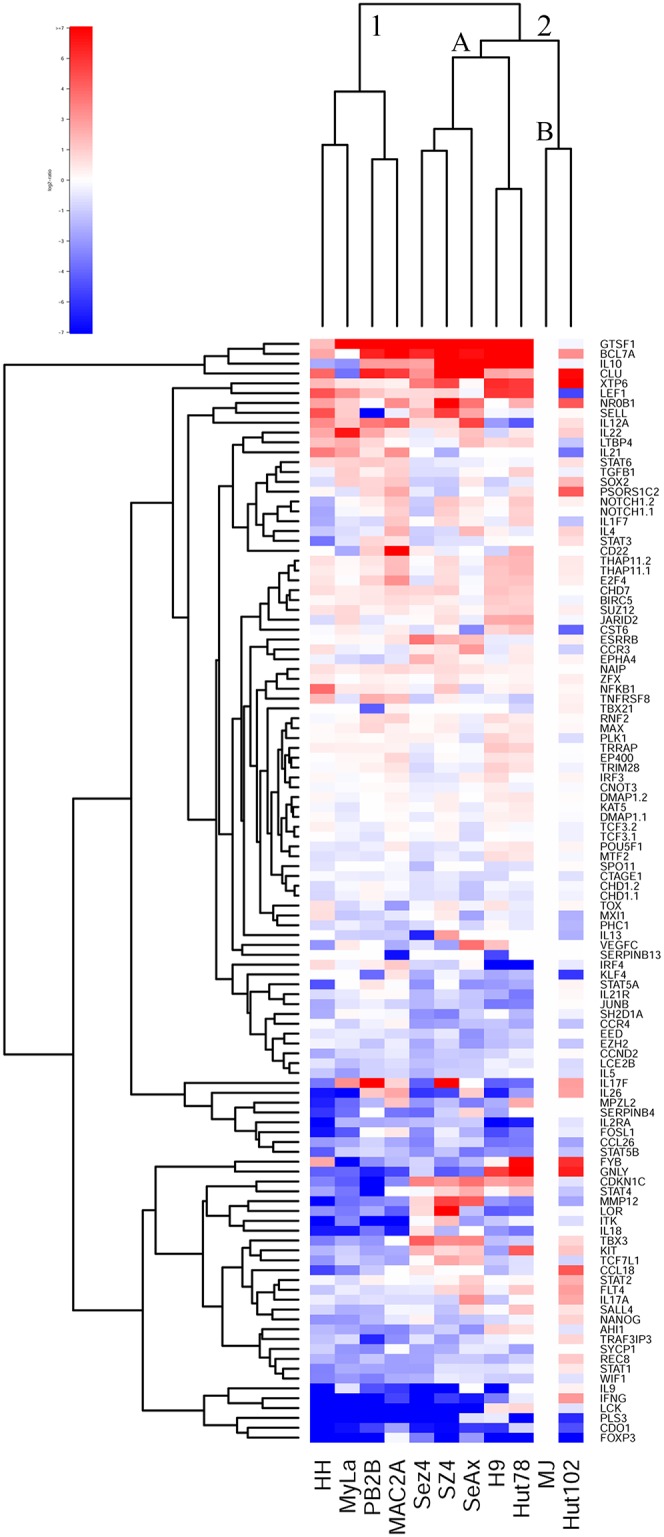
Unsupervised clustering analysis based on RT-PCR expression of 107 select genes in 11 patient-derived cell lines

This clustering analysis separated all cell lines into two clusters based on their gene expression profile (Figure 1). Cluster 1 included HH, MyLa, PB2B and Mac2A cells. Importantly, as highlighted in Supplementary Table 1/Supplementary Materials of this report, all these cells were derived from MF patients, albeit most of them had a leukemic stage IV disease. Also, notably, within this cluster Mac2A and PB2B cells clustered together based on the similarities of their gene expression profiles. This is expected, since, as described above, these cells are derived from the same clone that produced different variants of CTCL within the same patient.

Cluster 2, on the other hand, contained all cell lines that were derived from Sézary Syndrome patients (Sez4, SZ4, SeAx, H9 and Hut78) and *HTLV-1^+^* cells (MJ and Hut 102). Notably, these *HTLV-1^+^* cells lines clustered together in cluster 2B and away from the other SS cells that together formed cluster 2A. Hut78 and H9 clustered together based on their gene expression patterns, as expected. Similarly, Sez4 and SZ4 cells clustered together and they were very similar to the SeAx cells based on the expression of the tested genes.

Hence, this clustering analysis confirms that MyLa, PB2B, Mac2A and HH represent Mycosis Fungoides on a molecular level, while SeAx, Sez4, SZ4, H9 and Hut78 represent Sézary Syndrome. While, MJ and Hut102 *HTLV-1^+^* cells based on gene expression appear similar to the SS and not leukemic MF variant of CTCL, as highlighted by the karyotype analyses they truly do not represent either.

### Analysis of expression of specific poor vs. favorable markers in patient-derived CTCL cells

**Figure 2:**
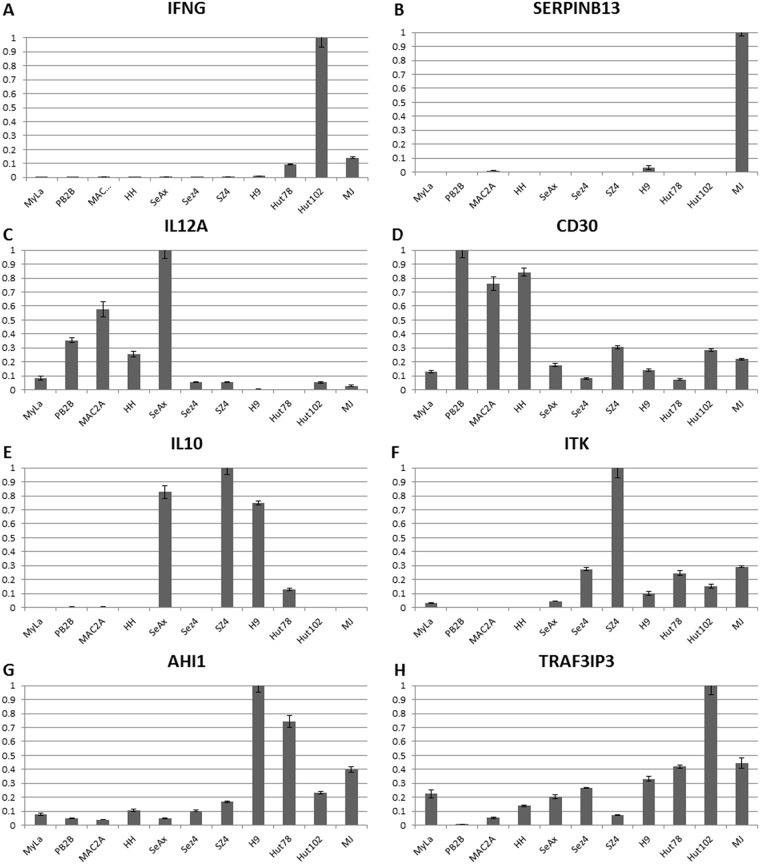
Individual gene expression findings for Th1 cytokines (IFNG, IL-12A), T reg cytokine (IL-10), CD30 and advanced disease/poor prognosis genes (ITK, AHI1 and TRAF3IP3) in 11 patient-derived cell lines **(A-C)** CTCL favorable prognosis genes IFNG, IL12A and SERPINB13 mRNA expression. **(D-H)** Poor prognosis genes CD30, IL-10, ITK, AHI1 and TRAF3IP3 expression.

Further in-depth analysis of gene expression across the 11 cell lines highlights important downregulation of IFN-γ in MF/SS cells (Figure 2A). Also, SERPINB13 favorable prognosis marker was downregulated in all cell lines (Figure 2B), therefore, pointing to the overall advanced disease nature of these cells. Notably, IL-12 is a key cytokine involved in promoting the Th1 immune response. CTCL is known to undergo a switch from the Th1 to Th2 immune response in advanced disease [19]. IL-12A was strongly expressed in MF cell lines (HH, MyLa, PB2B and Mac2A), but not in Sézary cell lines (Sez4, SZ4, Hut78 or H9) with a notable exception of SeAx (Figure 2C). None of the cells expressed IL-12B (data not shown) and, hence, are not able to produce a functional IL-12 cytokine. These findings point to the advanced disease state of all tested cells, but suggest that MF cells incompletely lost their ability to express some of the components of the IL-12 machinery. Also, importantly, CD30 expression was detected in all cell lines, but was strongest in PB2B, Mac2A cells that were derived from a known CD30^+^ MF/ALCL, as well as in HH cells that were also derived from an advanced MF patient (Figure 2D).

**Figure 3:**
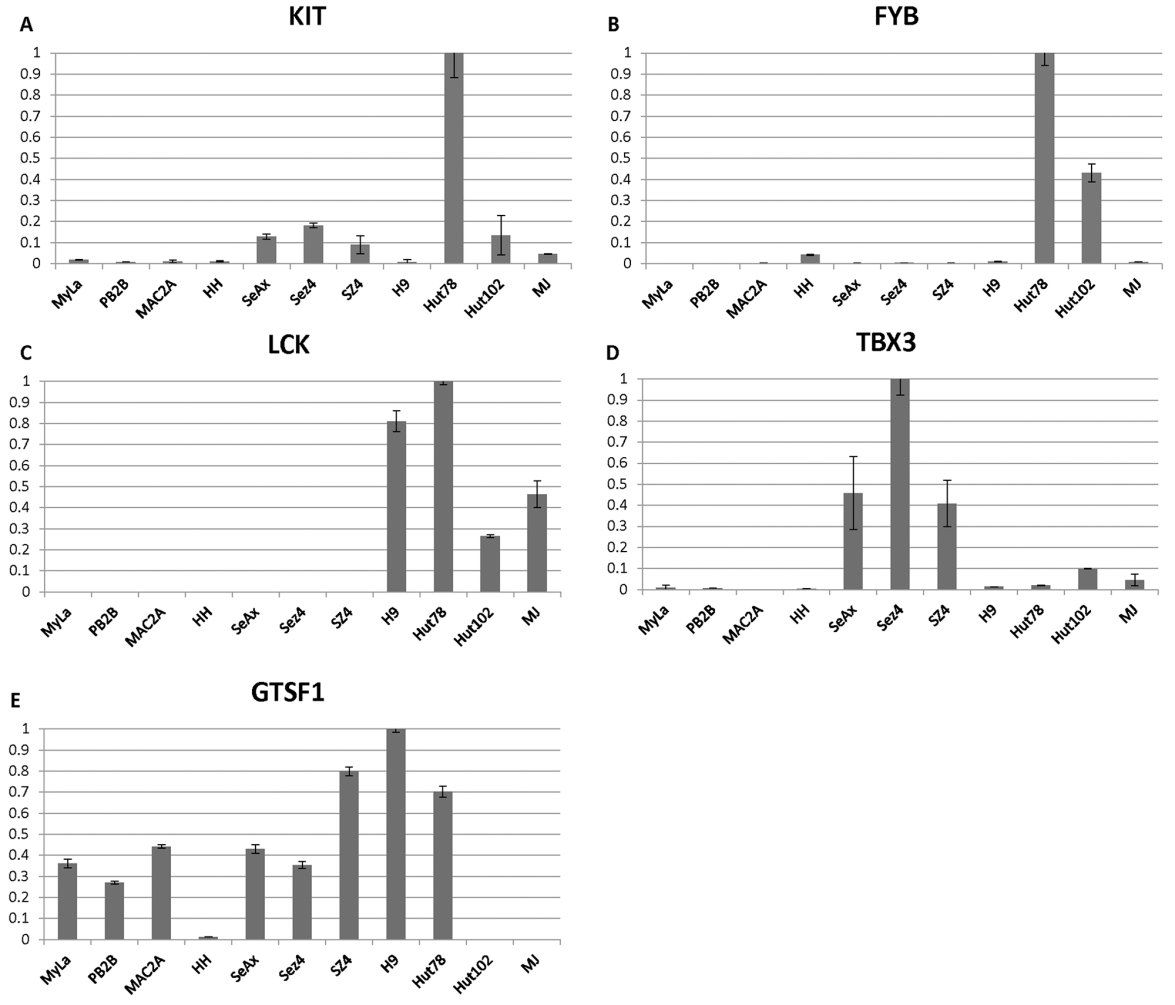
**(A-D)** Individual gene expression findings for advanced disease/poor prognosis genes (KIT, FYB, LCK, TBX3) and **(E)** cancer-testis poor prognosis gene GTSF1 in 11 patient-derived cell lines.

On the other hand, IL-10 a T reg cytokine that is known to be expressed in advanced stages was undetectable in MF cells MyLa, HH, Mac2A and PB2B, but was expressed across a panel of Sézary cells, but not *HTLV-1*^+^ cells (Figure 2E). Expression for a number of additional poor prognosis markers including ITK, AHI1, TRAF3IP, FYB, KIT, LCK and TBX3 was further upregulated preferentially in Sézary cells (Figures 2F-2H and Figure 3A-3D). GTSF1 poor prognosis marker cancer-testis gene was previously shown to be strongly expressed in MF/SS patients [18, 20, 44, 47]. This gene was strongly expressed in all 9 tested CTCL cell lines and was only weakly expressed in *HTLV-1^+^* cells.

These combined gene expression findings further corroborate our earlier conclusions and highlight that while all cells lines represent advanced CTCL disease, MyLa, PB2B/Mac2A, and HH represent Mycosis Fungoides, where PB2B, Mac2A and HH represent CD30^+^ MF and/or ALCL disease. On the other hand, SeAx, Sez4/SZ4, Hut78/H9 represent Sézary syndrome. Hut102 and MJ cells show gene expression profile that is similar to Sézary Syndrome, but truly represent a leukemic form of ATLL.

### TP53 mutation status in cell lines

TP53 mutations were previously reported to occur in advanced disease stages and were associated with poor survival in patients [51]. Hence, we performed the sequencing of the TP53 gene and identified that Sézary cells Hut78 and SeAx carry mutations of p53 (Table 2). HH, advanced MF line, had a change that affects the splicing of the gene. The most common codon polymorphism (at 72^nd^ codon) was Arg/Arg. Only MJ cells had a Pro/Arg polymporphism. MLPA analysis demonstrated duplication of the gene in MyLa and HH cells (Table 2).

**Table 2:** TP53 mutation status in patient-derived CTCL cell lines

Cell line	TP53 Status					
	cDNA		Protein		Codon 72	MLPA
	Allele 1	Allele 2	Allele 1	Allele 2		
**MyLa**	c.75-80 C>G	+	+	+	Arg/Arg	Dup x1-11
**Mac2A**	c.75-80 C>G	+	+	+	Arg/Arg	Normal
**PB2B**	c.75-80 C>G	+	+	+	Arg/Arg	Normal
**HH**	c.560-1G>A	c.560-1G>A	Splicing affected	Splicing affected	Arg/Arg	Del x1-11/+
**SeAx**	c.733G>A	c.733G>A	p.Gly245Ser	p.Gly245Ser	Arg/Arg	Normal
**SZ4**	c.75-80 C>G	c.75-80 C>G	+	+	Arg/Arg	Normal
**Sez4**	c.75-80 C>G	c.75-80 C>G	+	+	Arg/Arg	Normal
**Hut78**	c.75-80 C>G	c.75-80 C>G	p.Arg196X	p.Arg196X	Arg/Arg	Normal
	c.586 C>T	c.586 C>T				
**H9**	c.75-80 C>G	c.75-80 C>G	+	+	Arg/Arg	Normal
**MJ**	c.75-80 C>G	+	+	+	Pro/Arg	Normal
**Hut102**	c.75-80 C>G	+	+	+	Arg/Arg	Normal

**Table 3:** Sensitivity (IC50) of the tested cell lines to the 3 commonly used systemic therapies in CTCL

A		B		C	
Romidepsin		Vorinostat		Bexarotene	
Cells	IC50 (μM)	Cells	IC50 (μM)	Cells	IC50 (μM)
**Sez4**	**0.14**	**Hut78**	**1.43**	**MJ**	**2.62**
**SeAx**	**0.14**	**H9**	**2.29**	**SeAx**	**9.92**
**H9**	**0.16**	**PB2B**	**4.90**	**Sez4**	**11.35**
**MyLa**	**0.26**	**SeAx**	**5.79**	**PB2B**	**13.03**
**HH**	**0.41**	HH	29.59	**HH**	**13.89**
**Mac2A**	**0.48**	SZ4	30.30	MyLa	18.23
**PB2B**	**0.73**	MyLa	34.38	H9	23.72
**Hut78**	**0.77**	MJ	34.45	Hut102	28.06
MJ	2.97	Sez4	37.44	Mac2A	28.75
SZ4	3.03	Mac2A	>40	SZ4	28.87
Hut102	>4	Hut102	>40	Hut78	35.10

Hut78 and its clonally derived variant, H9, had a nonsense mutation of p53 in exon 6. SeAx cells had a deleterious Gly245Ser mutation in exon 7. HH cells harbor a c560-1G>A mutation, which is predicted to affect splicing in intron 5 and lead to partial loss of function. Many cell lines had a silent c75–80C>G polymorphism in intron 2, which is predicted not to affect gene expression or function (Table 2).

These findings are consistent with the previous reports of TP53 mutations in advanced disease patients. Considering that all 9 CTCL cell lines represent advanced stages of CTCL, it is not surprising that a number of these cells (i.e., Hut78, H9, SeAx and HH) have mutated TP53. Notably, *HTLV-1*^+^ MJ and Hut102 cells had wild type TP53.

### Testing cell line sensitivities to commonly used medication treatments in CTCL

Stage IIB-IVB MF patients that were refractory to two or more standard therapies demonstrated overall response rate of 45% or 55% with daily doses of bexarotene 300 or 650 mg/m^2^, respectively [52]. Histone deacetylase inhibitors (HDACs) on the other hand demonstrated partial response in 29.7% of patients with only 1 complete response for Vorinostat and 36% response rate including 5 complete responses for Romidepsin [52]. Hence, we wanted to subject our cell lines to these treatments at various concentrations to determine their sensitivities (i.e., IC50) to Romidepsin, Vorinostat and Bexarotene. As demonstrated in Table 3, the 11 cell lines demonstrated different sensitivities to the tested treatments. Interestingly SeAx and PB2B cells were relatively sensitive to all three treatments, Hut78 and H9 were sensitive to both HDAC inhibitors (i.e., Romidepsin and Vorinostat), while *HTLV-1*^+^ Hut102 and MJ cells were relatively resistant to the HDAC inhibitors at concentrations ≤40 μM.

### Study of patient–derived cells in NOD.Cg-Prkdc^scid^ Il2rg^tm1Wjl^/SzJ mice

**Figure 4:**
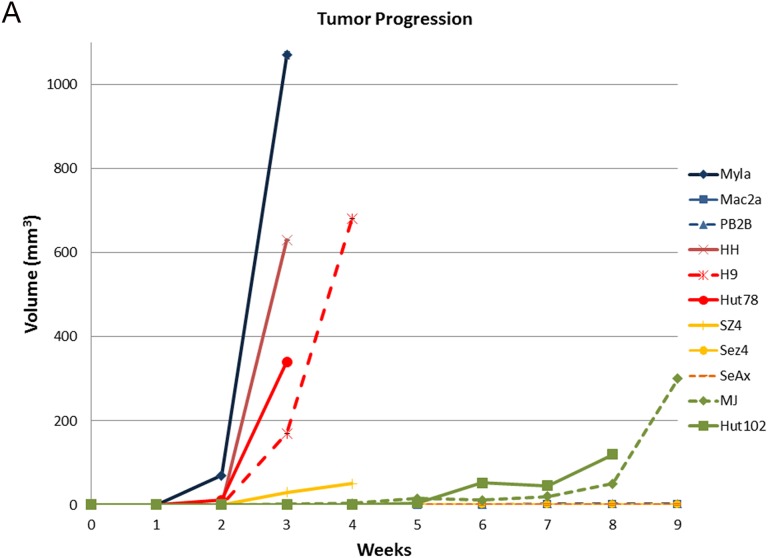

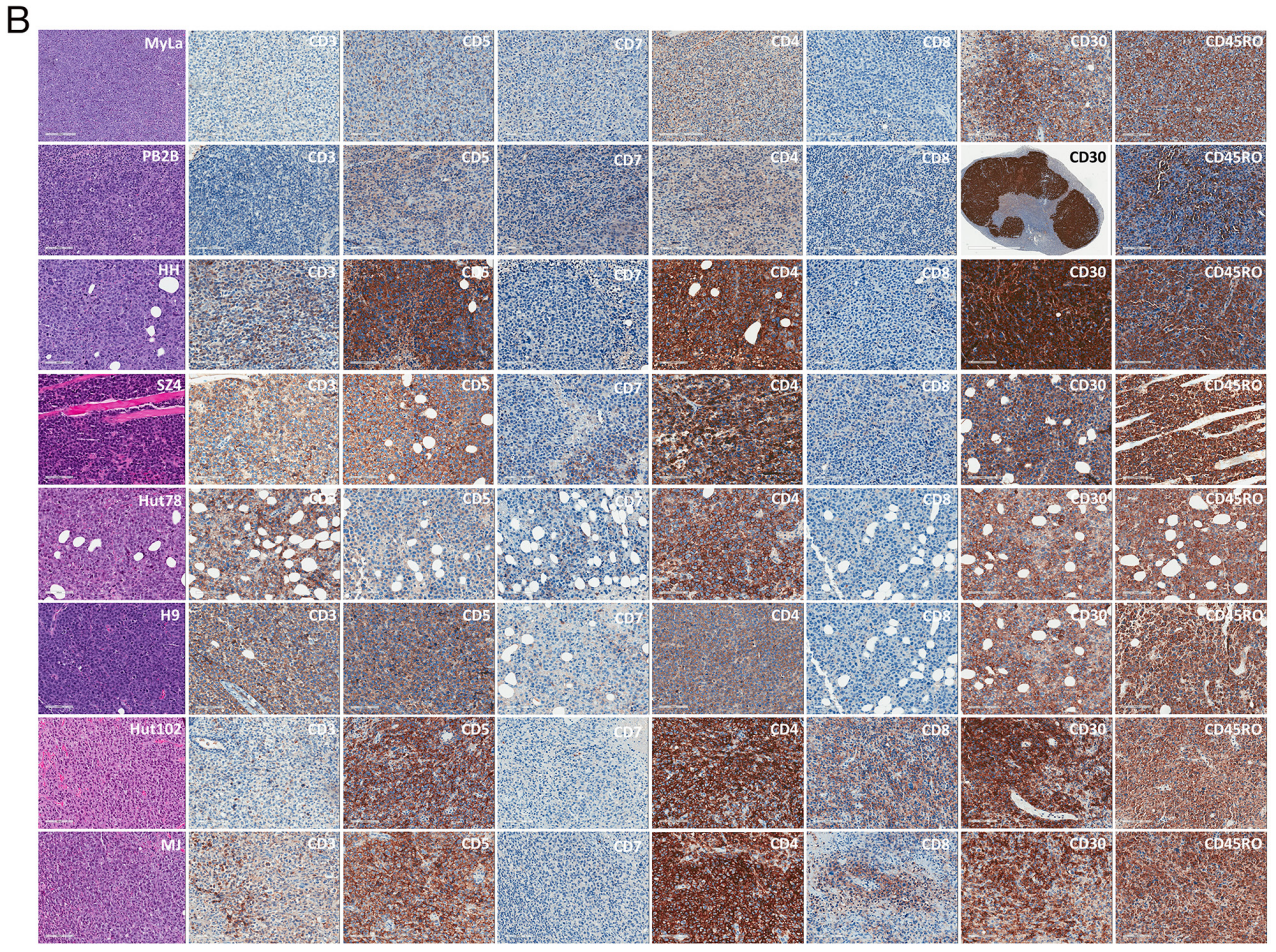
Evaluating the ability of patient-derived CTCL cell lines to produce xenograft tumors in *NOD.Cg-Prkdc^scid^ Il2rg^tm1Wjl^/SzJ* mice **(A)** Tumor growth measurements for 11 cell lines. Note, SZ4 mice had to be euthanized because they were barbed and piloerected even though the tumors did not reach 1 cm^3^ in volume. PB2B cells produced very small tumors that were heavily infiltrated by polymorphonuclear leukocytes. Sez4, SeAx and Mac2A cells did not produce tumors on multiple implantation attempts. **(B)** Hematoxylin and eosin staining of the obtained tissues and immunohistochemical characterization of these tumors based on pan T-cell marker expression (CD3, CD5, CD7); CD4 *vs.* CD8 expression; CD30 (advanced disease) and CD45RO (memory T-cell) marker expression.

We then wanted to extend our molecular observations and correlate our findings clinically by implanting and growing these cell lines as subcutaneous xenograft tumors in NOD.Cg-Prkdc^scid^ Il2rg^tm1Wjl^/SzJ (also commonly referred to as NSG strain) mice. Previous reports demonstrated that these mice could serve as a powerful model to study CTCL pathogenesis [53]. As demonstrated in Figure 4A, 8/11 cell lines were able to produce tumors in mice when implanted subcutaneously. Mac2A, Sez4 and SeAx cells failed to produce tumors on multiple attempts, while PB2B sporadically produced small tumors in 2/6 implantation attempts that were heavily infiltrated by polymorphonuclear leukocytes. Other 8 cell lines reliably produced subcutaneous tumors. Interestingly, MyLa and HH MF lines as well as H9 and Hut78 Sézary cells were most aggressive and produced sizable tumors within 2-4 weeks post implantation. Interestingly, SZ4, but not Sez4 cells also produced smaller tumors and at week 4 these mice had to be euthanized as they were found to be piloerected and barbed. Hut102 and MJ cells too produced measurable tumors, but only 7-9 weeks after the implantation of these cells.

The obtained xenograft tumors were analyzed in a similar way as human CTCL skin biopsy specimens. We performed Hematoxylin and Eosin (H&E) staining of the tumors, immunophenotyping based on CD3, CD4, CD5, CD7, CD8, CD30 and CD45 RO staining (Figure 4B) and T cell receptor (TCR) clonality studies using PCR (Table 4). H&E staining in all cases revealed atypical lymphocytes with many mitotic figures. All cells stained positive for CD4 and only *HTLV-1*^+^ cells had a concomitant positive staining for CD8. CD7 pan T cell marker expression was lost in all tumors, while CD3 and CD5 demonstrated variable expression patterns (Figure 4B). CD5 was expressed in all cells, while CD3 was not expressed in MyLa and PB2B cells and stained only occasional cells in Hut102 tumors. Other cells demonstrated moderate-to-strong CD3 staining (Figure 4B). CD30 was expressed in all cells. Notably PB2B tumors had regional/clonal expression pattern of this genes as islands of cells did not express CD30. CD45RO, a memory T cell marker, was also strongly expressed in all cell lines, as expected (Figure 4B).

**Table 4:** T-cell receptor beta and gamma clonality findings for the obtained xenograft tumor tissues

Cell line	TCR gamma receptor		TCR beta receptor		
	Vy-Jp	Vy-Jy	TCRB-A	TCRB-B	TCRB-C
MyLa	---	+++	---	---	---
PB2B	+++	---	---	---	---
HH	+++	+++	---	+	---
Hut78	---	+++	---	---	+++
H9	+++	+++	---	---	+++
Hut102	---	+++	---	---	---
MJ	---	+++	---	---	---

TCR gene rearrangement studies on these xenograft tumors documented that all tissues were derived from clonal events (Table 4). The RT-PCR analysis documented TCR gamma chain was clonal in all cell lines that produced tumors and in addition to that the TCR beta chain was also clonal in HH, Hut78 and H9 cells (Table 4).

## DISCUSSION

While the *HTLV-1* viruses and their ability to cause ATLL was discovered in 1980’s, still today, the controversy ensues whether *HTLV-1* plays any role in the pathogenesis of MF/SS even in the smallest subset of patients from *HTLV-1* endemic regions. Furthermore, critical biological differences between *HTLV-1*-driven ATLL and classic MF/SS variants of CTCL remain poorly defined. This study for the first time highlights robust critical molecular differences between *HTLV-1*^+^ patient-derived MJ and Hut102 cells that represent ATLL and classic *HTLV-1*^-^ MF/SS cells. Consistent with the well-described clinical observation for leukemic ATLL, these cells behave like advanced stage IV MF/SS cancers with respect to gene expression profiling and ability to produce tumors in mice. Furthermore, immunohistochemical analyses of cluster differentiation expression markers and TCR clonality studies in these tumors fall well within the expected range for the advanced MF/SS disease. Also, even though these cells showed lower sensitivity to HDAC inhibitors, their responses to tested medications were comparable to advanced MF/SS cells.

However, most importantly, the karyotype of these cells documented only minimal number of non-specific chromosomal alterations and these cells were mostly diploid. These findings are consistent with the small numbers of chromosomal abnormalities that were reported in ATLL patients [29, 30]. Also, a 10q24 leukemic chromosomal aberration, common in MF/SS, was characteristically absent in these cells.

Hence, like horses and zebras *HTLV-1^+^* leukemia cells and SS/leukemic MF cells look similar despite having a very different genetic composition/origin. This work highlights that the lack of multiple structural abnormalities (including the commonly seen 10q24 aberration) is a important difference between ATLL and classic MF/SS lymphomas that could be used as an important diagnostic test in these patients. In addition, our results indicate that *HTLV-1* virus is not involved in the pathogenesis of classic MF/SS since, as exemplified by the MJ/Hut102 cells, this virus drives a very different pathway of lymphomagenesis that does not lead to an accumulation of a substantial number of chromosomal alterations and, hence, produces a very different cancer on a genetic/chromosomal level. Hence, detection of *HTLV-1*^+^ serology in any patient should alert a physician that they are dealing with a fundamentally different cancer than the classic MF/SS irrespective of their clinical presentation and disease course.

In this study we also performed a comprehensive comparison and characterization of the available 9 classic MF/SS cell lines. Many researchers in the field use these cells and, unfortunately, often their choice of cells is driven by availability as opposed to understanding what diseases these cell lines truly represent. While some skeptics see limited value in these cells, our work highlights that these cell lines, in fact, do represent typical MF/SS patients. They represent classic karyotypic and gene expression heterogenetiy that is seen in MF/SS cancers. This is especially exemplified by the comparison of karyotype changes that were observed in the 9 studied cell lines when compared to MF/SS patients (Supplementary Table 4). On the molecular level they show similar gene expression patterns that we and others have extensively documented in advanced MF/SS patients [44–47, 50, 54]. As seen in a subset of advanced CTCL patients, a number of these cells carry deleterious *TP53* gene mutations. They also show heterogeneity of clinical responsiveness to Bexarotene and HDAC inhibitors that is commonly observed in advanced MF/SS disease. Importantly, a number of these cells are aggressive enough to produce tumors in NSG mice and these tumors show cluster differentiation marker staining results that are typically seen in classic MF/SS tumors.

We wish to highlight one important limitation in relation to these and other immortalized cancer cell lines. While the presented findings appear to be robust, it is always important to consider that molecular changes can differ in cell lines based on their passage number and culturing conditions.

In this work we further clarified that Sez4 and SZ4 genetically represent the same cell line. We also confirmed that the same malignant clone caused skin lesions in the patient that gave rise to Mac2A and PB2B cells. We validated that H9 and Hut78 cells represent the same clinical case/event, as expected. Importantly, based on our combined results presented in this paper, these 9 cell lines are only suitable to study advanced MF/SS disease stages. None of these cell lines represent early stage (≤IIA) mycosis fungoides. Notably, MyLa represent advanced skin MF, Mac2A/PB2B represent advanced skin CD30^+^ MF/ALCL, while HH represent leukemic CD30^+^ MF. SeAx, Sez4/SZ4 and Hut78/H9 represent true Sézary Syndrome. Hence, based on this study, researchers should use appropriate cell lines in their clinical/translational investigations.

In conclusion, this work highlights key genetic/biologic similarities and differences between *HTLV-1*^+^ and *HTLV-1*^-^ CTCL variants and provides extensive characterization for these 11 cell lines and places them in the context of the clinical spectrum for CTCL disease.

## MATERIALS AND METHODS

### Cell lines and culture/treatment conditions

HH, H9, Hut78, MJ and Hut102 patient-derived CTCL cell lines were previously described [55, 56] and were purchased from the American Tissue Culture Collection (ATCC). H9 is a clonal derivative of Hut78 cell line [57]. MyLa, PB2B, Mac2A, SZ4, SeAx, Sez4 were a generous gift from professors K. Kaltoft and N. Ødum (Copenhagen, Denmark) and were initially described elsewhere [31, 58–61]. Detailed summary of cell lines is provided in Supplementary Table 1. MJ, Hut78 cells were serially passaged in IMDM media (Invitrogen) containing 20% and 10% fetal bovine serum, respectively (FBS) (Invitrogen). HH, H9, Hut102, MyLa, Mac2A and SZ4 cells were grown in RPMI media containing 10% FBS. Finally, Sez4 and SeAx cells were grown in RPMI media containing 10% FBS, 5 ng/mL of recombinant human IL-2 and IL-4 (R&D Systems, Minneapolis, MN). All cells were grown in 5% CO_2_, 95% air humidified incubator at 37°C.

To inhibit histone deacetylase (HDAC) activity cells were treated with 0-40 μM of Suberoylanilide Hydroxamic Acid (SAHA also known as Vorinostat, Santa Cruz, Dallas, TX) or 0-4μM Romidepsin (Adooq Bioscience, Irvine CA). Cells were also treated with 0-40μM of Bexarotene (Santa Cruz Biotechnology, Dallas, TX).

### Quantitative RT-PCR analysis of gene expression

mRNA from cell lines was isolated using a Quiagen kit (Valencia, CA) and was converted into cDNA using Bio-Rad iScript cDNA synthesis kit. Gene expression on mRNA level was evaluated using quantitative RT-PCR as previously described [18, 19, 20, 43–45]. We have tested 107 genes that were studied and described in our prior work on CTCL patients [44, 50]. Primer pair sequences for the tested genes and control housekeeping genes are listed in Supplementary Table 5. Primer pairs for *Tax, Gag, Pol, Env* and *pX* HTLV-1 genes are also listed in Supplementary Table 5. The expression was standardized using genorm method [62] utilizing *ACTB, SDHA* and *YWHAZ* housekeeping genes. Gene expression in MJ cells was set as 1 fold similarly to the protocol in our previous studies [20, 45]. MJ cell line was selected as reference, since all genes showed detectable expression in this line. qRT-PCR relative expression was transformed as base-2 log of the ratio against MJ cells, independently for each gene. These log2-ratio data points were hierarchically clustered (euclidian distance, complete linkage) using both genes and cell lines as observation points to obtain two dendrograms. The log2-ratio data matrix is shown as a heatmap where rows (genes) and columns (cell lines) are ordered to match the respective dendrogram. Data and figures generated using the R project for statistical computing (https://www.r-project.org).

### G band and spectral karyotyping, TP53 sequencing and MTT cell survival analyses

G-band and spectral karyotyping of chromosomes was performed in our laboratory and by The Centre for Applied Genomics, The Hospital for Sick Children (Toronto, ON) as previously described [63].

TP53 sequencing was performed by the Molecular Genetics Laboratory, The Hospital For Sick Children (Toronto, ON) as previously described [64, 65]. MTT assay reagents were obtained from Sigma-Aldrich and were performed as previously described [66]. Briefly, standard curves were generated for each cell line to enable conversion from absorbance to cell number values. To obtain IC50 values, cells were plated in 96 well plates and treated for 24 hours using respective concentrations of SAHA (i.e, Vorinostat), Romidepsin and Bexarotene and percent kill was determined using standard MTT protocol.

### Xenograft model of CTCL

The University of Ottawa Animal Care and Ethics Committee approved all experimental procedures involving the xenograft experiments on the immunodeficient NOD.Cg-*Prkdc^scid^ Il2rg^tm1Wjl^*/SzJ (also commonly referred to as NSG) strain of mice (Jackson Laboratory, Bar Harbor, ME). These mice lack mature T cells, B cells or functional NK cells and are deficient in cytokine signaling. The CTCL cell lines Myla, Mac2a, PB2B, HH, H9, Hut78, SZ4, Sez4, SeAx, MJ and Hut102 were harvested individually from tissue culture, pelleted and re-suspended in DPBS (Corning, Manassas, VA). Eight week old female mice were injected subcutaneously (s.c) with 2.5×10^6^ cells suspended in a total volume of 200μl of sterile DPBS into the two hind flanks. Mice were housed with enrichment in groups of 4 in a biohazard facility. The condition of the mice was monitored daily for the first 48 hours post injection. Tumor onset and growth was then assessed on a weekly basis by flank palpation within a biosafety cabinet under aseptic conditions. As the tumors progressed, they were measured by slide calipers and recorded over the course of 9 weeks. When tumor progression neared the endpoint volume of 1cm^3^, the animals were monitored daily. At experiment termination or wellness endpoint, the mice were euthanized by CO_2_ followed by cervical dislocation and tumors were excised, measured and weighed.

Tissue processing, Hematoxylin and Eosin (H&E) staining, immunohistochemical analyses for CD3, CD4, CD5, CD7, CD8, CD30, CD45RO expression and TCR clonality analyses were subsequently performed on the formalin-fixed tumors as per standard clinical protocols in our hospital as previously described [67].

## SUPPLEMENTARY MATERIALS FIGURES AND TABLES













## Abbreviations

ATLL: adult T-cell leukemia/lymphoma
ATCC: American Tissue Culture Collection
BSA: body surface area
CTCL: cutaneous T-cell lymphomas
H&E: hematoxylin and eosin
HDACs: histone deacetylase inhibitors
HTLV-1: human T-cell lymphotropic virus type 1
LyP: lymphomatoid papulosis
MF: mycosis fungoides
cALCL: primary cutaneous anaplastic large cell lymphoma
SS: Sézary syndrome
TCR: T cell receptor
