# Supplementary material for: Analysis of CTCL cell lines reveals important differences between mycosis fungoides/Sézary syndrome vs. HTLV-1+ leukemic cell lines
Source: Oncotarget. 2017 Oct 7;8(56):95981–98. doi: 10.18632/oncotarget.21619 (PMC5707075; doi:10.18632/oncotarget.21619)
Supplement: Supplementary file 1 [file oncotarget-08-95981-s001.pdf]

Analysis of CTCL cell lines reveals important differences between mycosis fungoides/Sézary syndrome *vs. HTLV-1*$^+$ leukemic cell lines

SUPPLEMENTARY MATERIALS

Detailed description of patients, who donated their tissues to establish cell lines

MyLa cells were derived from an 82-year-old Caucasian male with 80% Body Surface Area (BSA) involvement by MF with extensive lymphadenopathy (clinical stage IIA disease). Cell line was established form a skin biopsy at that time. Patient eventually developed progressive disease and, unfortunately, died. Autopsy only showed dermatopahic lymphadenopathy and no internal organ involvement [1].

Mac2A cells were obtained from a 47-year-old Caucasian male initially presenting with Lymphomatoid Papulosis (LyP) in 1971, concomitantly developing Hodgkin's disease in 1975. He then developed extensive CTCL lesions in 1983, which were believed to be within the LyP-ALCL CD30$^+$ lymphoproliferative disorder disease spectrum, and in 1985-1987 he was observed to have erythroderma and Sézary cells in blood and developed ulcerating tumors. In 1988 autopsy documented extensive CD30$^+$ ALCL in retroperitoneal lymph nodes [2]. Interestingly, it was proven that his Hodgkin's disease, LyP and CTCL/ALCL originated form the same T cell clone [2]. Mac2A and PB2B cells were established from this patient at different times. Mac2A was established from a rapidly growing skin tumor in 1987 at the time when this patient had documented Sézary cells circulating in blood, while PB2B cell line was established "at a later aggressive stage form a skin nodule showing large cell lymphoma"[3, 4]. Hence, clinically both cell lines represent advanced aggressive CD30$^+$ cutaneous lymphoma.

HH cells were obtained from a 61-year-old Caucasian male who had tumor stage (IIB) MF with lymph node disease and later progressed to leukemic MF (clinical stage IVB) with involvement of the spleen. Cell line was established from peripheral blood just prior to patient's death [5]. Blood smear analysis at the time showed ~20% of cells with deeply convoluted nuclei [5].

SZ4 cells were obtained from a 66-year-old African-American female initially diagnosed with stage IIA MF in 1984, who rapidly progressed and developed lymph node involvement with diffuse architectural effacement (clinical stage IVA_2) and erythroderma shortly after in 1985. Blood involvement was diagnosed at that time and cell line was established form a peripheral blood sample. SZ4 and Sez4 cells are derived from the same Sézary patient. Based on the literature search it appears that, inadvertently, at some point the name of this cell line has changed from SZ4 to Sez4 [6, 7], thus, creating some confusion in the field.

Hut78 cells were obtained from peripheral blood of a 53-year-old Caucasian male with Sézary Syndrome involving "skin, blood, lymph nodes and liver." H9 cell line is a clone of Hut78 that was selected for permissiveness of *HIV-1* replication, and has been used to isolate and propagate *HIV-1* from the blood of patients with acquired immunodeficiency syndrome (AIDS) [8].

SeAx cells were derived from a peripheral blood of a 66-year-old female with a 8-year history of exfoliative erythroderma and palmoplantar keratoderma with a diagnosis of Sézary Syndrome involving the bone marrow. Importantly, no prior MF plaques were reported in this patient. Lymph node biopsy revealed only dermatopathic changes. This cell line requires IL-2 for continuous growth [9].

MJ and Hut102 cells were isolated from patients, who clinically at the time were diagnosed with "Mycosis Fungoides variant of CTCL", as described in the papers [10, 11], and were later found to harbor *HTLV-1*. In these papers it was not stated whether the diagnosis was changed to ATLL. Hut102 cells were established from a lymph node biopsy [10], while MJ cells were obtained from peripheral blood [10, 11].

REFERENCES

1. Kaltoft K, Bisballe S, Dyrberg T, Boel E, Rasmussen PB, Thestrup-Pedersen K. Establishment of two continuous T-cell strains from a single plaque of a patient with mycosis fungoides. *In Vitro* Cell Dev Biol. 1992; 28A:161-167.
2. Davis TH, Morton CC, Miller-Cassman R, Balk SP, Kadin ME. Hodgkin's disease, lymphomatoid papulosis, and cutaneous T-cell lymphoma derived from a common T-cell clone. N Eng J Med. 1992; 326:1115-1122.
3. Wasik MA, Seldin DC, Butmarc JR, Gertz R, Marti R, Maslinski W, Kadin ME. Analysis of IL-2, IL-4 and their receptors in clonally-related cell lines derived from a patient with a progressive cutaneous T-cell lymphoproliferative disorder. Leuk Lymphoma. 1996; 23:125-136.
4. Zhang Q, Nowak I, Vonderheid EC, Rook AH, Kadin ME, Nowell PC, Shaw LM, Wasik MA. Activation of Jak/STAT proteins involved in signal transduction pathway mediated

by receptor for interleukin 2 in malignant T lymphocytes derived from cutaneous anaplastic large T-cell lymphoma and Sezary syndrome. Proc Natl Acad Sci U S A. 1996; 93:9148-9153.

5. Starkebaum G, Loughran TP Jr, Waters CA, Ruscetti FW. Establishment of an IL-2 independent, human T-cell line possessing only the p70 IL-2 receptor. Int J Cancer. 1991; 49:246-253.
6. Lin WM, Lewis JM, Filler RB, Modi BG, Carlson KR, Reddy S, Thornberg A, Saksena G, Umlauf S, Oberholzer PA, Karpova M, Getz G, Mane S, et al. Characterization of the DNA copy-number genome in the blood of cutaneous T-cell lymphoma patients. J Invest Dermatol. 2012; 132:188-197.
7. Abrams JT, Lessin S, Ghosh SK, Ju W, Vonderheid EC, Nowell P, Murphy G, Elfenbein B, DeFreitas E. A clonal CD4-positive T-cell line established from the blood of a patient with Sezary syndrome. J Invest Dermatol. 1991; 96:31-37.
8. Mann DL, O'Brien SJ, Gilbert DA, Reid Y, Popovic M, Read-Connole E, Gallo RC, Gazdar AF. Origin of the HIV-susceptible human CD4+ cell line H9. AIDS Res Hum Retroviruses. 1989; 5:253-255.
9. Kaltoft K, Bisballe S, Rasmussen HF, Thestrup-Pedersen K, Thomsen K, Sterry W. A continuous T-cell line from a patient with Sézary syndrome. Arch Dermatol Res. 1987; 279:293-298.
10. Poiesz BJ, Ruscetti FW, Gazdar AF, Bunn PA, Minna JD, Gallo RC. Detection and isolation of type C retrovirus particles from fresh and cultured lymphocytes of a patient with cutaneous T-cell lymphoma. Arch Proc Natl Acad Sci U S A . 1980; 77:7415-7419.
11. Popovic M, Sarin PS, Robert-Gurroff M, Kalyanaraman VS, Mann D, Minowada J, Gallo RC. Isolation and transmission of human retrovirus (human t-cell leukemia virus). Science. 1983; 219:856-859.

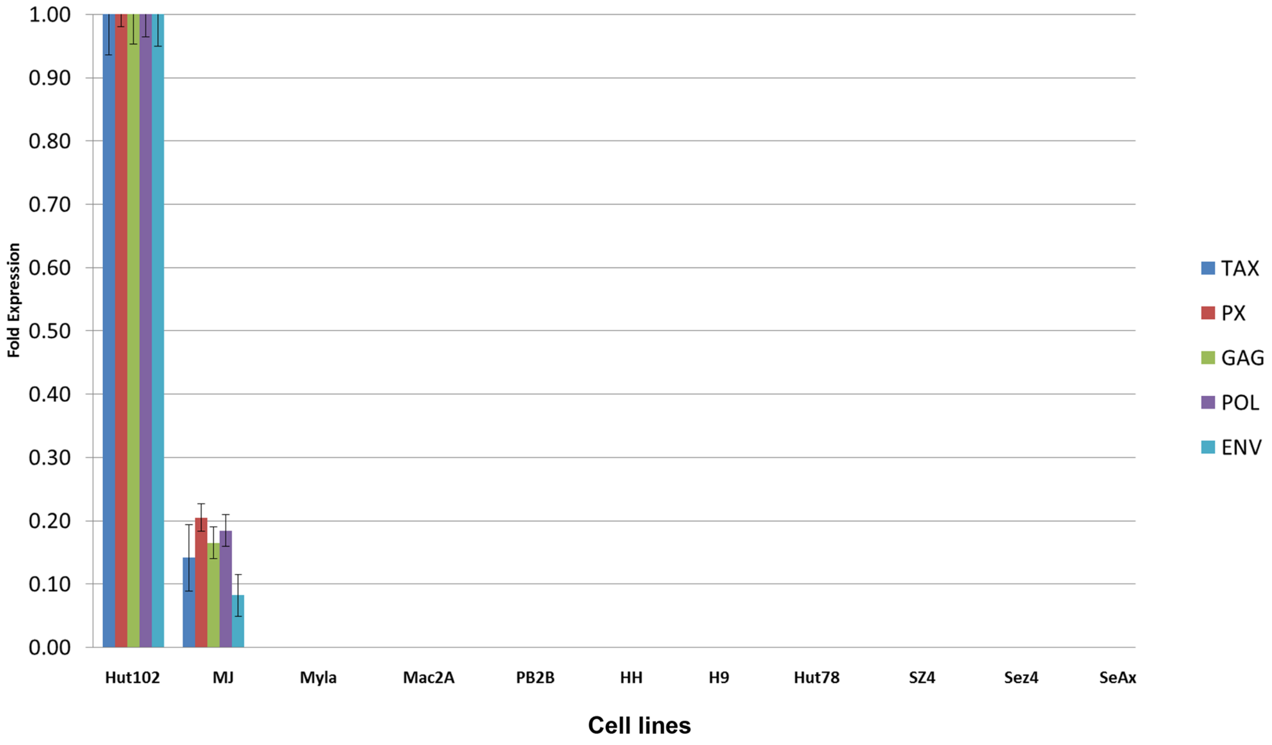

Supplementary Figure 1: RT-PCR expression of *Tax, Gag, Pol, Env* and *pX* genes to identify which cells harbor *HTLV-1* virus.

Supplementary Table 1: Summary of commonly used CTCL cell lines for research.

See Supplermentary File 1

Supplementary Table 2: Clonal abnormalities that occurred in all cells analyzed from a given cell line are highlighted in yellow. Clonal abnormalities or breakpoints that were in common between multiple cell lines are highlighted in green. Data presented by cell line.

See Supplermentary File 2

Supplementary Table 3: Clonal abnormalities or breakpoints that were in common between multiple cell lines are highlighted in green. Data presented by chromosome.

See Supplermentary File 3

Supplementary Table 4: Comparison of structural chromosomal aberrations between the studied CTCL cell lines and patient findings reported in literature based on 15 selected studies [1–15].

See Supplermentary File 4

Supplementary Table 5: Primers used for RT-PCR experiments.

See Supplermentary File 5
