# Supplementary material for: Analysis of CTCL cell lines reveals important differences between mycosis fungoides/Sézary syndrome vs. HTLV-1+ leukemic cell lines
Source: Oncotarget. 2017 Oct 7;8(56):95981–98. doi: 10.18632/oncotarget.21619 (PMC5707075; doi:10.18632/oncotarget.21619)
Supplement: Supplementary file 2 [file oncotarget-08-95981-s002.docx]

| **Cell Line** | **Information on patients, who donated the cells for research** | **Tissue source** | **Year established** | **Reference** |
| --- | --- | --- | --- | --- |
| MyLa | 82-year-old Caucasian male with 80% Body Surface Area (BSA) involvement by Mycosis Fungoides with extensive lymphadenopathy (stage IIA). Cell line was established form a skin biopsy at that time. Patient eventually developed progressive disease leading to patient’s death. Autopsy only showed dermatopahic lymphadenopathy and no internal organ involvement. | Skin Biopsy | 1990 | [[1](#_ENREF_1)] |
| Mac2A | 47-year-old Caucasian male initially presenting with Lymphomatoid Papulosis (LyP) in 1971, concomitantly developing Hodgkin’s disease in 1975. He the developed primary cutaneous ALCL in 1983 and in 1985-1987 he was observed to have erythroderma and Sézary cells in blood and developed ulcerating tumors. In 1988 autopsy documented extensive CD30^+^ ALCL in retroperitoneal lymph nodes. | Skin Tumor | 1987 | [[2](#_ENREF_2)] |
| PB2B | 47-year-old Caucasian male (same patient as described above for Mac2A cells). This cell line was established “at later aggressive stage form skin nodule showing large cell lymphoma”. | Skin Nodule | 1987-1988 | [[3](#_ENREF_3), [4](#_ENREF_4)] |
| HH | 61-year-old Caucasian male who had tumor stage (i.e., IIB) MF with lymph node disease and later progressed to leukemic MF (stage IVB) with spleen involvement. Cell line was established from peripheral blood just prior to patient’s death from his lymphoma. | Peripheral Blood | 1986 | [[5](#_ENREF_5)] |
| Hut78 | 53-year-old Caucasian male with Sézary Syndrome involving “skin, blood, lymph nodes and liver.” | Peripheral Blood | 1980 | [[6](#_ENREF_6)] |
| H9 | This cell lines is a clone of Hut78. The H9 clone was selected for permissiveness of *HIV-1* replication, and has been used to isolate and propagate *HIV-1* from the blood of patients with acquired immunodeficiency syndrome (AIDS) and pre-AIDS conditions. | Peripheral Blood | 1983 | [[7](#_ENREF_7)] |
| SZ4 | 66-year-old African-American female initially diagnosed with MF stage IIA in 1984 and then rapidly developed lymph node involvement with diffuse architectural effacement and erythroderma shortly after (in 1985). Blood involvement was diagnosed at that time and cell line was established form a peripheral blood. SZ4 and Sez4 cells are derived from the same patient. | Peripheral Blood | 1986 | [[8](#_ENREF_8)] |
| Sez4 | Sz4 and Sez4 cells are derived from the same patient. Inadvertently, at some point the name of this cell line in literature was changed from SZ4 to Sez4 [[9](#_ENREF_9)]. | Peripheral Blood | 1986 | [[8](#_ENREF_8), [9](#_ENREF_9)] |
| SeAx | 66-year-old female with exfoliative erythroderma and palmoplantar keratoderma with a diagnosis of Sézary Syndrome involving bone marrow. No prior MF plaques were reported in this patient. Cell line requires IL-2 for continuous growth. | Peripheral Blood | 1987 | [[10](#_ENREF_10)] |
| Hut102 | 28 year old African-American male with Cutaneous T-Cell Lymphoma described as “mycosis fungoides” with skin lesions and lymph node disease. The cell line harbors *HTLV-1* virus. | Lymph Node | 1978 | [[11](#_ENREF_11)] |
| MJ | 50-year-old Caucasian male with “Mycosis Fungoides from Boston, MA”. The cell line harbors *HTLV-1* virus. | Peripheral Blood | 1982 | [[12](#_ENREF_12)] |

**Supplementary Table 1.** Summary of commonly used CTCL cell lines for research.

**References**:

1. Kaltoft K, Bisballe S, Dyrberg T, Boel E, Rasmussen PB and Thestrup-Pedersen K. Establishment of two continuous T-cell strains from a single plaque of a patient with mycosis fungoides. In vitro cellular & developmental biology : journal of the Tissue Culture Association. 1992; 28A(3 Pt 1):161-167.

2. Davis TH, Morton CC, Miller-Cassman R, Balk SP and Kadin ME. Hodgkin's disease, lymphomatoid papulosis, and cutaneous T-cell lymphoma derived from a common T-cell clone. The New England journal of medicine. 1992; 326(17):1115-1122.

3. Wasik MA, Seldin DC, Butmarc JR, Gertz R, Marti R, Maslinski W and Kadin ME. Analysis of IL-2, IL-4 and their receptors in clonally-related cell lines derived from a patient with a progressive cutaneous T-cell lymphoproliferative disorder. Leukemia & lymphoma. 1996; 23(1-2):125-136.

4. Zhang Q, Nowak I, Vonderheid EC, Rook AH, Kadin ME, Nowell PC, Shaw LM and Wasik MA. Activation of Jak/STAT proteins involved in signal transduction pathway mediated by receptor for interleukin 2 in malignant T lymphocytes derived from cutaneous anaplastic large T-cell lymphoma and Sezary syndrome. Proceedings of the National Academy of Sciences of the United States of America. 1996; 93(17):9148-9153.

5. Starkebaum G, Loughran TP, Jr., Waters CA and Ruscetti FW. Establishment of an IL-2 independent, human T-cell line possessing only the p70 IL-2 receptor. International journal of cancer. 1991; 49(2):246-253.

6. Gazdar AF, Carney DN, Bunn PA, Russell EK, Jaffe ES, Schechter GP and Guccion JG. Mitogen requirements for the in vitro propagation of cutaneous T-cell lymphomas. Blood. 1980; 55(3):409-417.

7. Mann DL, O'Brien SJ, Gilbert DA, Reid Y, Popovic M, Read-Connole E, Gallo RC and Gazdar AF. Origin of the HIV-susceptible human CD4+ cell line H9. AIDS research and human retroviruses. 1989; 5(3):253-255.

8. Abrams JT, Lessin S, Ghosh SK, Ju W, Vonderheid EC, Nowell P, Murphy G, Elfenbein B and DeFreitas E. A clonal CD4-positive T-cell line established from the blood of a patient with Sezary syndrome. The Journal of investigative dermatology. 1991; 96(1):31-37.

9. Lin WM, Lewis JM, Filler RB, Modi BG, Carlson KR, Reddy S, Thornberg A, Saksena G, Umlauf S, Oberholzer PA, Karpova M, Getz G, Mane S, Garraway LA, Dummer R, Berger CL, et al. Characterization of the DNA copy-number genome in the blood of cutaneous T-cell lymphoma patients. The Journal of investigative dermatology. 2012; 132(1):188-197.

10. Kaltoft K, Bisballe S, Rasmussen HF, Thestrup-Pedersen K, Thomsen K and Sterry W. A continuous T-cell line from a patient with Sezary syndrome. Archives of dermatological research. 1987; 279(5):293-298.

11. Poiesz BJ, Ruscetti FW, Gazdar AF, Bunn PA, Minna JD and Gallo RC. Detection and isolation of type C retrovirus particles from fresh and cultured lymphocytes of a patient with cutaneous T-cell lymphoma. Proceedings of the National Academy of Sciences of the United States of America. 1980; 77(12):7415-7419.

12. Popovic M, Sarin PS, Robert-Gurroff M, Kalyanaraman VS, Mann D, Minowada J and Gallo RC. Isolation and transmission of human retrovirus (human t-cell leukemia virus). Science. 1983; 219(4586):856-859.
