# Supplementary material for: Analysis of CTCL cell lines reveals important differences between mycosis fungoides/Sézary syndrome vs. HTLV-1+ leukemic cell lines
Source: Oncotarget. 2017 Oct 7;8(56):95981–98. doi: 10.18632/oncotarget.21619 (PMC5707075; doi:10.18632/oncotarget.21619)
Supplement: Supplementary file 3 [file oncotarget-08-95981-s003.docx]

| Cell lines | | | | | | | | | | | Clonal chromosome abnormalities that are common between cell lines |
| --- | --- | --- | --- | --- | --- | --- | --- | --- | --- | --- | --- |
| MyLa | HH | Hut78 | H9 | PB2B | Mac2A | SeAx | Sez4 | SZ4 | Hut102 | MJ |  |
|  |  |  |  |  |  | SeAx | Sez4 | SZ4 |  |  | -X |
|  | HH | Hut78 | H9 | PB2B | Mac2A |  |  |  |  |  | -Y |
|  |  | Hut78 | H9 |  |  |  |  |  |  |  | t(X;13)(p11.2~11.4;q14),der(2)(2pter→2q21::2?q31→2?q33::8q24.1→8qter), der(3)(3pter→3q29::10q24→10qter), ?4, der(4)(4pter→4p?14::4?q21→4?p14::4?q25→4?q21::16p?11.1→16p?13.3::13q14→13qter), t(5;6)(p10;p10), +der(6)(4qter→4q21~24::6?p21→6?q13::6?q23→6qter),  der(7)(10?→10?::7p14~15→7qter)x2, der(9)(Y?→Y?::9p21~22→9qter), der(10)(10pter→10q24:), der(10)t(7;10)(q11.2;q22.2~22.3), ?del(14)(q?11.2q?24), -16, +17, +der(19)t(19;20)(q13.?3;q11.2), der(19;22)(q10;q10)x2, der(20)(:9p11→9p24::20p13→20qter), der(20)(20pter→20q10::20?→20?::20q12→20qter), der(21)(11qter→11q13~14::21q10→21qter) |
|  |  |  |  |  |  |  | Sez4 | SZ4 |  |  | psu dic(1;16)(16pter→16q24::1q22~25→1q10::1q10→1qter), derivative chromosome 1 segments 3pter→3p21::?→?::3p21→3p13~14::1p31 and 1q42::8p21→8pter, t(1;11;8)(q42;q23;p21) or its derivative chromosome(s), der(3)t(3;6)(q12~13;?), der(5)(5pter→5p10::5q35→5q31::1?→1?::7?→7?::5q35→5q31:), der(6)(6pter→6p21.3::6p21.1→6q23~24::1p34→1pter), t(6;11)(q16~21;q21~22), -7, breakpoint 7q22, der(9)t(9;14)(p13~22;q13)x2, der(10)(10pter→10q24:), der(11)(:14q?32→14q?24::11p15→11q21~22:), der(13)(:13q?31→13q?14::13p12→13qter), -14, der(15)(10qter→10q24::15p12→15qter), del(16)(q11.1), i(17)(q10), der(18)t(8;18)(q11.2;p11.3), der(19)t(7;19)(p11;q13.4), der(21)t(9;21)(p22;q22)x2, +der(?)(acro-p::8?q13→8?q22:) |
| MyLa | HH | Hut78 |  | PB2B | Mac2A |  |  |  |  | MJ | Breakpoint 2p11.2 with three copies of segment 2pterp11.2. HH has uncertain three copies of segment 2pterp?14~21. Hut78 has four or uncertain six copies of segments from 2p. MyLa has three copies of segment 2pterp13. These six cell lines have gain in 2p relative to each of its ploidy level. |
|  | HH |  |  | P2B |  | SeAx |  |  |  |  | Breakpoint 3q24 |
|  | HH | Hut78 | H9 |  |  |  |  |  |  |  | Contain complex rearrangements of chromosome 4 with chromosome 4 segments with uncertain or unknown breakpoints present. |
| MyLa |  |  |  | P2B | Mac2A |  |  |  |  |  | Breakpoint 6q15. Mac2A had del(6)(q15)[5], MyLa had del(6)(q15q23)[5], and P2B had der(5;6)(5pter→5p10::6q10→6q15:)[5]. All three cell lines possibly had loss of segment 6q15q23 relative to each of its ploidy level. |
|  |  |  |  | P2B | Mac2A |  |  |  |  |  | der(8)(?9?→?9?::8p22→8qter), del(12)(q11q13), der(15)t(2;15)(p11.2;p12), del(16)(q11.2q22), der(20)(:20?q13.1→20?q13.1::20p13→20q?13.1::20q?13.1→20qter), breakpoint 22q10 |
|  |  | Hut78 | H9 |  |  |  | Sez4 | SZ4 |  |  | Breakpoint 9p22 |
|  | HH | Hut78 | H9 |  |  | SeAx | Sez4 | SZ4 |  |  | Breakpoint 10q24 and/or der(10)(10pter→10q24:) |
|  |  | Hut78 | H9 |  |  |  |  |  |  |  | Breakpoint 11q13~14 |
|  |  |  |  |  |  | SeAx |  |  |  |  | Breakpoint 11q14~21 |
|  |  | Hut78 |  |  |  |  |  |  |  |  | Breakpoint 11q21 |
|  |  |  |  |  | Mac2A |  |  |  |  |  | Breakpoint 11q22 |
|  |  |  |  |  |  |  | Sez4 | SZ4 |  |  | Breakpoints 11q21~22 and 11q23 |
|  | HH |  |  |  | Mac2A | SeAx |  |  |  |  | Breakpoint 12q21. Mac2A and SeAx had gain of segment 12q21qter relative to each of its ploidy level. |
|  |  | Hut78 | H9 |  |  | SeAx |  |  |  |  | -13 |
| MyLa | HH |  |  |  | Mac2A |  |  | SZ4 |  |  | Breakpoint 14q13 |
| MyLa |  |  |  | PB2B |  |  |  |  |  |  | Breakpoint 14q22 |
|  | HH |  | H9 |  |  |  |  |  |  |  | -15 |
| MyLa |  | Hut78 | H9 |  |  |  |  |  |  |  | +17 |
|  | HH |  |  |  |  |  |  | SZ4 |  | MJ | Breakpoint 17q25 |
|  |  |  |  | PB2B |  |  | Sez4 | SZ4 |  |  | Breakpoint 18p11.3 |
| MyLa | HH |  |  | PB2B |  |  |  |  |  |  | Breakpoint 18q22 |
|  |  |  |  |  |  | SeAx | Sez4 |  |  |  | +20 |
| MyLa |  |  |  |  |  |  | Sez4 | SZ4 |  |  | Breakpoint 21q22 |

**Supplementary Table 2.** Clonal abnormalities that occurred in all cells analyzed from a given cell line are highlighted in yellow. Clonal abnormalities or breakpoints that were in common between multiple cell lines are highlighted in green. Data presented by cell line.
