# Supplementary material for: Analysis of CTCL cell lines reveals important differences between mycosis fungoides/Sézary syndrome vs. HTLV-1+ leukemic cell lines
Source: Oncotarget. 2017 Oct 7;8(56):95981–98. doi: 10.18632/oncotarget.21619 (PMC5707075; doi:10.18632/oncotarget.21619)
Supplement: Supplementary file 4 [file oncotarget-08-95981-s004.docx]

| **Cell line name** | **Modal chr # <ploidy>** | **Ploidy status** | **Expected gender** | **X** | **Y** | **1** | **2** | **3** | **4** | **5** | **6** | **7** | **8** | **9** | **10** | **11** | **12** | **13** | **14** | **15** | **16** | **17** | **18** | **19** | **20** | **21** | **22** | **M** |
| --- | --- | --- | --- | --- | --- | --- | --- | --- | --- | --- | --- | --- | --- | --- | --- | --- | --- | --- | --- | --- | --- | --- | --- | --- | --- | --- | --- | --- |
| SZ4 | 79~81 <4n> | Hypotetraploid | Female | XX,-X |  | psu dic(1;16) der(1) with chr 3 and/or chr 8 involvement der(1)t(1;11;8) |  | der(3)t(3;6) |  | der(5) with chr 1 and 7 involvement | der(6) with chr 1 involvement t(6;11) | -7 Breakpoint 7q22 | der(8)t(1;11;8) | Breakpoint 9p22 der(9)t(9;14)x2 | Breakpoint 10q24 and der(10)(10pter->10q24:) | der(11) with chr 14 involvement der(11)t(1;11;8) Breakpoints 11q21~22 and 11q23 |  | der(13) | -14 Breakpoint 14q13 | der(15)t(10;15) | del(16)(q11.1) | i(17)(q10) Breakpoint 17q25 | der(18)t(8;18)(q11.2;p11.3) Breakpoint 18p11.3 | der(19)t(7;19) |  | der(21)t(9;21)x2 Breakpoint 21q22 |  | +der(?) |
| Sez4 | 77~80 <3n> | Hypertriploid | Female | XX,-?X |  | psu dic(1;16) der(1) with chr 3 and/or chr 8 involvement der(1)t(1;11;8) |  | der(3)t(3;6) |  | der(5) with chr 1 and 7 involvement | der(6) with chr 1 involvement t(6;11) | -7 Breakpoint 7q22 | der(8)t(1;11;8) | Breakpoint 9p22 der(9)t(9;14)x2 | Breakpoint 10q24 and der(10)(10pter->10q24:) | der(11) with chr 14 involvement der(11)t(1;11;8) Breakpoints 11q21~22 and 11q23 |  | der(13) | -14 | der(15)t(10;15) | del(16)(q11.1) | i(17)(q10) | der(18)t(8;18)(q11.2;p11.3) Breakpoint 18p11.3 | der(19)t(7;19) | +20 | der(21)t(9;21)x2 Breakpoint 21q22 |  | +der(?) |
| Hut78 | 71~75 <3n> | Hypertriploid | Male | t(X;13) | -Y |  | der(2) Gain of 2p (with different breakpoints) relative to ploidy level | der(3)t(3;10) | Complex rearrangements of chr 4 der(4) with chr 13 and chr 16 involvement | t(5;6) | +der(6) with chr 4 involvement | der(7)x2 |  | Breakpoint 9p22 der(9)t(Y;9) | Breakpoint 10q24 and der(10)(10pter->10q24:) der(10)t(7;10) | Breakpoint 11q13~14 Breakpoint 11q21 |  | -13 | ?del(14) |  | -16 | +17 |  | +der(19)t(19;20) der(19;22)x2 | der(20) with chr 9 involvement der(20) with chr 20 involvement, different morphology than Mac2A or P2B | der(21)t(11;21) |  |  |
| H9 | 60~70 <3n> | Hypertriploid | Male | t(X;13) | -Y |  | der(2) | der(3)t(3;10) | Complex rearrangements of chr 4 der(4) with chr 13 and chr 16 involvement | t(5;6) | +der(6) with chr 4 involvement | der(7)x2 |  | Breakpoint 9p22 der(9)t(Y;9) | Breakpoint 10q24 and der(10)(10pter->10q24:) der(10)t(7;10) | Breakpoint 11q13~14 |  | -13 | ?del(14) | -15 | -16 | +17 |  | +der(19)t(19;20) der(19;22)x2 | der(20) with chr 9 involvement der(20) with chr 20 involvement, different morphology than Mac2A or P2B | der(21)t(11;21) |  |  |
| SeAx | 64~71 <3n> | Hypertriploid | Female | X,-X |  |  |  | Breakpoint 3q24 |  |  |  |  |  |  | Breakpoint 10q24 and der(10)(10pter->10q24:) | Breakpoint 11q14~21 | Breakpoint 12q21. Gain of 12q21qter relative to ploidy level. | -13 |  |  |  |  |  |  | +20 |  |  |  |
| MyLa | 46~48 <2n> | Diploid | Male | X | Y |  | Gain of 2p (with different breakpoints) relative to ploidy level |  |  |  | Breakpoint 6q15 Possible loss of 6q15q23 relative to ploidy level |  |  |  |  |  |  |  | Breakpoint 14q13 Breakpoint 14q22 |  |  | +17 | Breakpoint 18q22 |  |  | Breakpoint 21q22 |  |  |
| Hut102 | 46 or 92 <4n> | Diploid and tetraploid | Male | X | Y |  |  |  |  |  |  |  |  |  |  |  |  |  |  |  |  |  |  |  |  |  |  |  |
| MJ | 45~47 <2n> | Hyperdiploid | Male | X | Y |  | Gain of 2p (with different breakpoints) relative to ploidy level |  |  |  |  |  |  |  |  |  |  |  |  |  |  | Breakpoint 17q25 |  |  |  |  |  |  |
| PB2B | 45 <2n> | Hypodiploid | Male | X | -Y |  | Gain of 2p (with different breakpoints) relative to ploidy level | Breakpoint 3q24 |  |  | Breakpoint 6q15 Possible loss of 6q15q23 relative to ploidy level |  | der(8) with chr 9 involvement |  |  |  | del(12)(q11q13) |  | Breakpoint 14q22 | der(15)t(2;15) | del(16)(q11.2q22) |  | Breakpoint 18p11.3 Breakpoint 18q22 |  | der(20) with chr 20 involvement, different morphology than H9 or Hut78 |  | Breakpoint 22q10 |  |
| Mac2A | 43~44 <2n> | Hypodiploid | Male | X | -Y |  | Gain of 2p (with different breakpoints) relative to ploidy level |  |  |  | Breakpoint 6q15 Possible loss of 6q15q23 relative to ploidy level |  | der(8) with chr 9 involvement |  |  | Breakpoint 11q22 | del(12)(q11q13) Breakpoint 12q21. Gain of 12q21qter relative to ploidy level. |  | Breakpoint 14q13 | der(15)t(2;15) | del(16)(q11.2q22) |  |  |  | der(20) with chr 20 involvement, different morphology than H9 or Hut78 |  | Breakpoint 22q10 |  |
| HH | 44~45 <2n> | Hypodiploid | Male | X | -Y |  | Gain of 2p (with different breakpoints) relative to ploidy level | Breakpoint 3q24 | Complex rearrangements of chr 4 |  |  |  |  |  | Breakpoint 10q24 and der(10)(10pter->10q24:) |  | Breakpoint 12q21 |  | Breakpoint 14q13 | -15 |  | Breakpoint 17q25 | Breakpoint 18q22 |  |  |  |  |  |

**Supplementary Table 3.** Clonal abnormalities or breakpoints that were in common between multiple cell lines are highlighted in green. Data presented by chromosome.
