# Supplementary material for: Analysis of CTCL cell lines reveals important differences between mycosis fungoides/Sézary syndrome vs. HTLV-1+ leukemic cell lines
Source: Oncotarget. 2017 Oct 7;8(56):95981–98. doi: 10.18632/oncotarget.21619 (PMC5707075; doi:10.18632/oncotarget.21619)
Supplement: Supplementary file 5 [file oncotarget-08-95981-s005.docx]

|  | **Chromosomal aberrations observed in patient-derived cell lines** | |  |  |  |  | **Chromosomal aberrations observed in MF/SS patients** |  |  |
| --- | --- | --- | --- | --- | --- | --- | --- | --- | --- |
| **Chromosome** | **Region** | **Structural Change** | **Cell Line(s)** | **Cells (out of 5 cells examined )** |  | **Region** | **Structural Change** | **CTCL Variant** | **Reference** |
| **1** | 1 (overall) | 3 normal copies (+1) | Hut78 | 5 |  |  |  |  |  |
|  | p10 | Translocation (unknown 2?); 1pterp10 (2 copies of segment) | SeAx | 3 | 1 |  |  |  |  |
|  | p21 | Segment (1p21q21) | SeAx | 5 |  | p21.2 | Loss of 1p21.1p13.1 in stage IIB patient | MF | [1] |
|  |  |  |  |  |  | p21 | Loss of 1p21p31 in skin samples | MF | [2] |
|  | p31 | Translocation | Sez4/SZ4 | 5 |  | p31 | Loss of 1p21p31 in skin samples | MF | [2] |
|  | p32-34 | Translocation | SZ4 | 5 |  | p32 | Inversion of 1p32q11 | SS | [3] |
|  |  |  |  |  |  | p32 | Translocation with 17q25 | SS | [4] |
|  |  |  |  |  |  | p33 | Diminished 1p33p36 | SS | [3] |
|  |  |  |  |  |  | p33 | Loss of 1p33p36 in 50% of patients | MF | [5] |
|  | p34 | Translocation (two copies) | Sez4 | 5 |  | p33 | Reciprocal translocation with 4p16 | MF | [5] |
|  | p34.3-35 | Reciprocal translocation (balanced) | PB2B | 4 |  | p34.3-35 | Chromosomal loss 1p34.3-p35 in stage IIB patient | MF | [6] |
|  |  |  |  |  |  | p34 | Gain of 1p364 in 3 cases | SS | [7] |
|  |  |  |  |  |  | p34 | Loss of 1p34 in 1 case | SS | [7] |
|  | p35-36.1 | Translocation | HH | 5 |  | p36 | Loss of 1p36 (deletion) | MF | [3] |
|  |  |  |  |  |  | p36 | Deletion of 1p31p36 in 38% of patients | SS | [3] |
|  |  |  |  |  |  | p36 | Deletion of 1p36 | MF/SS | [7] |
|  |  |  |  |  |  | p36 | Loss of 1p36.33p36.22 in stage IIB patient | MF | [1] |
|  |  |  |  |  |  | p36.33 | Loss of 1p36.33p22.1 | SS | [8] |
|  |  |  |  |  |  | p36 | Translocation with 8q24 in stage IVB patient | SS | [1] |
|  |  |  |  |  |  | p36 | Add 1p36 in stage IV patient | SS | [1] |
|  |  |  |  |  |  | p36 | Gain of 1p36 | MF | [2] |
|  |  |  |  |  |  | p36 | Loss of 1p33p36 in 50% of patients | MF | [5] |
|  | p36.1 | Segment copy (4p16) | SeAx | 4 |  | p36 | Loss of 1p22-1p36 | MF | [5] |
|  | q10 | Pseudodicentric | Sez4/SZ4 | 5 |  |  |  |  |  |
|  | q10 | Segment (2 copies); 1q10ter (2 copies) | SeAx | 3 |  |  |  |  |  |
|  | q12-21 | Translocation | PB2B | 2 |  |  |  |  |  |
|  | q21 | Segment (1q21-1p36.1;1q21-1qter) | SeAx | 5 |  | q21 | Translocation with 19p13.3 | MF | [4] |
|  |  |  |  |  |  | q21 | Gain 1q21q41 | MF | [9] |
|  |  |  |  |  |  | q21 | Gain in 1q21q22 in 41% of patients | MF | [10] |
|  | q22-25 | Pseudodicentric | Sez4/SZ4 | 5 |  | q22 | Gain in 1q21q22 in 41% of patients | MF | [10] |
|  |  |  |  |  |  | q22 | Loss of 1q22qter in skin samples | MF | [2] |
|  |  |  |  |  |  | q25 | Loss of 1q25 | MF | [7] |
|  |  |  |  |  |  | q25 | Deletion of 1q25 | MF | [3] |
|  |  |  |  |  |  | q25 | Gain in 1q25q31 in 35% of patients | MF | [11] |
|  | q31 | Segment attached | H9 | 5 |  | q31 | Gain in 1q25q31 in 35% of patients | MF | [11] |
|  |  |  |  |  |  | q31 | Gain in 1q31q32 in 45% of patients | MF | [10] |
|  | q32 | Break | MyLa | 5 |  | q32 | Gain in 1q31q32 in 45% of patients | MF | [10] |
|  |  |  |  |  |  | q32 | Deleted in stage IV patient | SS | [1] |
|  | q36.2-36.3 | Translocation | MyLa | 5 |  | q42.12 | Loss of 1p21.2q42.12 in stage IIB patient | MF | [1] |
|  | q42 | Segment/break-reciprocal T | Sez4/SZ4 | 2, 5 |  | q42 | Deleted 1q42 | SS | [3] |
|  |  |  |  |  |  |  |  |  |  |
|  |  | Summary of structural aberrations seen in chromosome 1 across multiple cell lines: |  |  |  |  |  |  |  |
|  | p34: Sez4/SZ4 and PB2B | |  |  |  |  |  |  |  |
|  | p35: Pb2B and HH | |  |  |  |  |  |  |  |
|  | p36.1: HH and SeAx | |  |  |  |  |  |  |  |
|  | q10: Sez4/SZ4 and SeAx | |  |  |  |  |  |  |  |
|  | q21: PB2B and SeAx | |  |  |  |  |  |  |  |
|  |  |  |  |  |  |  |  |  |  |
| **2** | 2 (overall) | 4 normal copies (+2) | Sez4/SZ4 | 5 | **2** |  |  |  |  |
|  | Unknown | Whole arm translocation and long arm of chromosome 19 at centromere | SeAx | 3 |  |  |  |  |  |
|  | 2?2? | Translocation (1p10 ); (11q14-21) | SeAx | 3 |  |  |  |  |  |
|  | p10 | Whole arm translocation | Hut78 | 5 |  |  |  |  |  |
|  | p11.2 | Translocation | PB2B | 5 |  | p11.2 | Gain in 2p11.2 | SS | [12] |
|  | p11.2 | Translocation | Mac2A | 5 |  | p11.2 | Gain of 2p11.2 in MF patient lymph node biopsy | MF | [2] |
|  | p11.2 | Translocation | MJ | 5 |  |  |  |  |  |
|  | p13 | Translocation (3 copies) | MyLa | 5 |  | p13 | Translocation with 10q24 | SS | [4] |
|  |  |  |  |  |  | p13 | Translocation with 1p13 | MF | [4] |
|  | p?14-21 | Translocation | HH | 5 |  | p21 | Loss 2p21pter in stage IVB patient | SS | [6] |
|  |  |  |  |  |  | p21.3 | Loss of 2p21.3q34 | MF | [2] |
|  | ?p22-23 | Uncertain segments (2 copies) | Hut78 | 4 |  | p22 | Gain of 2p22 from MF patients-skin samples | MF | [2] |
|  |  |  |  |  |  | p22 | Diminished 2p22p24 | SS | [3] |
|  |  |  |  |  |  | p22 | Loss of 2p22pter in node samples | MF | [2] |
|  | p24 | Reciprocal translocation (balanced) | PB2B | 5 |  | p24 | Loss of 2p24 | MF | [7] |
|  |  |  |  |  |  | p24 | Gain of 2p24 | MF | [7] |
|  |  |  |  |  |  | p24 | Diminished 2p22p24 | SS | [3] |
|  | p25 | Segment (1 or 2 copies) | SeAx | 3 |  | p25 | Deletion of 2p21p25 | MF | [3] |
|  | q14.3-21 | Translocation | H9 | 5 |  |  |  |  |  |
|  | q21 | Segment (2 copies) | H9/Hut78 | 5 |  | q21 | Translocation with 12q24 | MF | [4] |
|  | q21?q31 | Deletion | H9 | 3 |  |  |  |  |  |
|  | ?q31-?q33 | Segment (2 copies) | H9/Hut78 | 5 |  | q31 | Gain in 2q24-q31 | SS | [9] |
|  | q3?2q3?3 | Uncertain deletion and reunion | HH | 5 |  |  |  |  |  |
|  | q32-33 | Translocation | MyLa | 5 |  | q32-33 | Loss of 2q32q33 in skin sample | MF | [2] |
|  | q33 | Translocation | H9 | 5 |  |  |  |  |  |
|  |  | Summary of structural aberrations seen in chromosome 2 across multiple cell lines: |  |  |  |  |  |  |  |
|  | p11.2: PB2B, Mac2A and MJ | |  |  |  |  |  |  |  |
|  | q33: Hut78/H9, MyLa | |  |  |  |  |  |  |  |
|  | Gain in 2p relative to ploidy: HH, Hut78, Mac2A, MJ, MyLa, PB2B | |  |  |  |  |  |  |  |
|  |  |  |  |  |  |  |  |  |  |
| **3** | 3 (overall) | Normal chromosomes (+1) | Sez4 |  | **3** |  |  |  |  |
|  | 3 (overall) | Short arm translocation between 3 and 17-fused at centromeres | SeAx | 5 |  |  |  |  |  |
|  | 3?3? | Segment | SeAx | 5 |  |  |  |  |  |
|  | p10 | Translocation | HH | 4 |  |  |  |  |  |
|  | p10 | Segment (17p10) | SeAx | 5 |  |  |  |  |  |
|  | p12-13 | Translocation | Sez4 | 5 |  | p12 | Translocation with unknown chromosome segment | SS | [4] |
|  |  |  |  |  |  | p12 | Enhanced 3p12q13 | SS | [3] |
|  |  |  |  |  |  | p12 | Loss of 3p11p12 | MF | [2] |
|  |  |  |  |  |  | p12.3 | Gain 3pterp12.3 in stage IIB patient | MF | [1] |
|  | p13-14 | Translocation | Sez4/SZ4 | 5 |  | p14 | Add 3p14 in IVB patient | SS | [1] |
|  |  |  |  |  |  | p14 | Enhanced 3p14p21 | SS | [3] |
|  |  |  |  |  |  | p21 | Enhanced 3p14p21 | SS | [3] |
|  | p21 | Translocation | Sez4/SZ4 | 5 |  | p21 | Translocation of 3p21 with 22q13 in stage IVB patient | SS | [1] |
|  | p25-26 | Translocation | HH | 5 |  | p25 | Gain of 3p25 in 71% of patients | MF/SS | [7] |
|  | q10 | Translocation | SZ4 | 2 |  | q10 | Gain 3q10q13 in stage IVB patient | SS | [6] |
|  | q11.2 | Translocation | HH | 5 |  |  |  |  |  |
|  | q12 | Segment | SeAx | 5 |  |  |  |  |  |
|  | q12 | Translocation (17q10) (2 copies) | SeAx | 5 |  |  |  |  |  |
|  | q13.2 | Translocation | H9 | 5 |  |  |  |  |  |
|  | q13.2-13.3 | Translocation | SZ4 | 3 |  |  |  |  |  |
|  | ?q13.3q21 | Uncertain copy of segment | SZ4 | 3 |  | q13 | Enhanced 3p12q13 | SS | [3] |
|  | q12-13 | Segment | Sez4 | 5 |  | q13 | Gain 3q10q13 in stage IVB | SS | [6] |
|  | q22-23 | Reciprocal translocation (balanced) | Mac2A | 5 |  | q21 | Chromosomal enhancement/gain 3q21q22 in stage IVB patient | SS | [6] |
|  | q23 | Translocation | Mac2A | 5 |  | q22 | Chromosomal enhancement/gain 3q21q22 in stage IVB patient | SS | [6] |
|  |  |  |  |  |  | q22 | Diminished 3q22q24 | MF | [3] |
|  | q23-24 | Translocation (Xp11.4-21) | SeAx | 4 |  | q23 | Loss of 3q23q26 | MF | [2] |
|  | q24 | Break | Mac2A | 2 |  | q24 | Diminished 3q22q24 | MF | [3] |
|  | q24 | Translocation | PB2B | 3 |  |  |  |  |  |
|  | q24 | Translocation | HH | 5 |  |  |  |  |  |
|  | q29 | Segment (two copies) | Hut78/H9 | X1(1), x2(4) |  | q29 | Diminished 3q29 | SS | [3] |
|  |  | Summary of structural aberrations seen in chromosome 3 across multiple cell lines: |  |  |  |  |  |  |  |
|  | p10: HH and SeAx | |  |  |  |  |  |  |  |
|  | q13.2: H9 and SZ4 | |  |  |  |  |  |  |  |
|  | q23: Mac2A and SeAx | |  |  |  |  |  |  |  |
|  | q24: SeAx, Mac2A, HH, PB2B | |  |  |  |  |  |  |  |
|  |  |  |  |  |  |  |  |  |  |
| **4** | Unknown | Copy segment from 4 | Hut78/H9 HH | 5 | **4** |  |  |  |  |
|  | p?14 | Translocation segment | Hut78/H9 | 5 |  |  |  |  |  |
|  | p15 | Translocation (10q25) | Sez4 | 5 |  |  |  |  |  |
|  | p16 | Translocation (1p36.1) | SeAx | 4 |  | p16 | Gain of 4p16-pter in MF patient skin sample | MF | [2] |
|  |  |  |  |  |  | p16.1 | Gain in 4p16.1 | SS | [12] |
|  | q10 | Pseudodicentric with break | SZ4 | 5 |  |  |  |  |  |
|  | q13 | Balanced reciprocal translocation (5q14) | MyLa | 5 |  |  |  |  |  |
|  | q21 | Breakpoint segment insertions (unknown 4q?q?) (1p35-36.11pter) | HH | 5 |  | q21 | Translocation with 13p11 | MF | [4] |
|  | q21-24 | Translocation segment (2 copies) | Hut78 | 5 |  |  |  |  |  |
|  | ?q21?p14 | Uncertain pericentric inversion | Hut78/H9 | 5 |  |  |  |  |  |
|  | ?q25?q21 | Uncertain pericentric inversion | Hut78/H9 | 5 |  |  |  |  |  |
|  | q22-24 | Segment (translocation with unk.6) | H9 | 2 |  |  |  |  |  |
|  | q23-27 | Translocation segment (7q22) | Sez4 | 5 |  | q27 | Deletion of 4q27 | SS | [4] |
|  | ?q25 | Translocation | H9 | 5 |  |  |  |  |  |
|  | q28 | Translocation | Sez4 | 3 |  | q28 | Loss of 4p11q28 | MF | [2] |
|  | q31 | Breakpoint Segment insertions (unknown 4q?q?) (1p35-36.11pter) | HH | 5 |  | q31 | Translocation with 12? |  | [13] |
|  |  |  |  |  |  | q31.3 | Chromosomal loss in 4q31.3qter in stage IVA patient | MF/SS | [6] |
|  |  |  |  |  |  | q31.23 | Loss of 4q31.23 | SS | [8] |
|  | q33 | Paracentric inversion | SZ4 | 5 |  | q33 | Chromosomal loss in 4q33qter in stage IVB patient | SS | [6] |
|  | q35 | Paracentric inversion | SZ4 | 5 |  |  |  |  |  |
|  |  | Summary of structural aberrations seen in chromosome 4 across multiple cell lines: |  |  |  |  |  |  |  |
|  | q21: HH and Hut78/H9 | |  |  |  |  |  |  |  |
|  | q23: H9 and Sez4 | |  |  |  |  |  |  |  |
|  |  |  |  |  |  |  |  |  |  |
| **5** | 5 (overall) | Trisomy | SZ4/Sez4 | 5 | **5** |  |  |  |  |
|  | 5 (overall) | Translocation: short arm of #5 and long arm of #6 fused at centromeres (5pter10, 6q10q15) B | PB2B | 5 |  |  |  |  |  |
|  | 5 (overall) | Translocation short arm fused at centromere with unknown short arm of arocentric chromosome | H9 | 2 |  |  |  |  |  |
|  | 5?5? | Translocation with #8 | Hut78 | 2 |  |  |  |  |  |
|  | 5?5? | Uncertain segment with 8, 9 | Mac2A | 4 |  |  |  |  |  |
|  | p10 | Translocation: short arms of #5 and #6 and long arms respectively and fused at centromeres | Hut78/H9 | x2 (5) |  |  |  |  |  |
|  | p10 | Translocation (6p10) (2 copies) | Hut78 | 5 |  |  |  |  |  |
|  | p10 | Segments (5pterp10,5q35q31, 1?1?, 7?7? And 5q35q31) | SZ4 | 4;5 |  |  |  |  |  |
|  | p13 | Translocation (8p22-23) (2 copies) | SeAx | 4 |  | p13 | Loss of 5p13q14 | MF | [2] |
|  | p13 | Translocation (10q24) (2 copies) | SeAx | 5 |  | p15.3 | Gain of 5p15.3pter in skin samples from patients | MF | [2] |
|  | p15 | Segments (10qterq24, 9q34q13 and 5p15pter) | HH | 5 |  | p15.33 | Gain of 5p15.33 | SS | [8] |
|  | q10 | Segment (6q10-6q15) | PB2B | 5 |  |  |  |  |  |
|  | q14 | Reciprocal translocation (4q13) | MyLa | 5 |  | q14.3 | Loss of 5q14.3 | SS | [12] |
|  |  |  |  |  |  | q14.3 | Loss of 5q12.1q14.3 in stage IIB patient | MF | [1] |
|  |  |  |  |  |  | q14 | Loss of 5q14q23.3; 5q14qter | MF | [2] |
|  |  |  |  |  |  | q14 | Diminished 5q14q35 | SS | [3] |
|  |  |  |  |  |  | q31 | Gain of 5p31qter from node sample | MF | [2] |
|  |  |  |  |  |  | q31 | Enhanced 5q31 | SS | [3] |
|  | q31 | Segments (5pterp10,5q35q31, 1?1?, 7?7? And 5q35q31) | SZ4/Sez4 | 4;5 |  | q31.2 | Gain of 5q23.3q31.2 (amplification) in stage IIB patient | MF | [1] |
|  | ?q33 | Translocation with unknown breakpoint (8q10-8q22-24.1); (20qter-20q10…8..) | Hut78 | 2 |  | q33 | Loss of 5q33.3q34 in stage IVA patient | SS | [1] |
|  | q35 | Uncertain segment attachment- Translocation | MyLa | 5 |  |  |  |  |  |
|  | q35 | Segments (5pterp10,5q35q31, 1?1?, 7?7? And 5q35q31) | SZ4/Sez4 | 4;5 |  | q35 | Diminished 5q14q35 | SS | [3] |
|  |  | Summary of structural aberrations seen in chromosome 5 across multiple cell lines: |  |  |  |  |  |  |  |
|  | p10: Hut78/H9 and SZ4 | |  |  |  |  |  |  |  |
|  | q35: MyLa and SZ4/Sez4 | |  |  |  |  |  |  |  |
|  |  |  |  |  |  |  |  |  |  |
| **6** | 6 (overall) | Trisomy | H9 | 5 | **6** |  |  |  |  |
|  | 6 (overall) | Short arm of #5 and long arm of #6 fused at centromeres (5pterp10, 6q10q15) | PB2B | 5 |  |  |  |  |  |
|  | 6?6? | Uncertain segment translocation with 3q12 | Sez4/SZ4 | 5 |  | 6q | Loss of 6q | MF | [2] |
|  | 6?6? | Uncertain segment translocation | SeAx | 5 |  |  |  |  |  |
|  | 6?6? | Uncertain segment (4pter-4q22-24::?6?-?6?) | H9/Hut78 | 2, 5 |  |  |  |  |  |
|  | 6?6? | Uncertain segment-Translocation (10q10) | PB2B | 5 |  |  |  |  |  |
|  | p10 | Segment (6p21.1p10) | Sez4 | 2 |  |  |  |  |  |
|  | p10 | Translocation (5p10) | H9/Hut78 | x2(4); x2(5) |  |  |  |  |  |
|  | p12 | Translocation (1811.3) | PB2B | 5 |  |  |  |  |  |
|  | p21 | Segments (?p21q13) (2 copies) | H9/Hut78 | 5 |  | p21 | Gain of 6p21 from skin sample from patient | MF | [2] |
|  | p21.1 | Segment (6p21.1q23-24) (2 copies) | Sez4/SZ4 | 2, 5 |  |  |  |  |  |
|  | p21.1-21.2 | Translocation (22q11.2) | Mac2A | 5 |  |  |  |  |  |
|  | p21.3p21.1 | Deletion of segment | Mac2A | 5 |  | p21.32 | Gain of 6p21.32 | SS | [8] |
|  | p21.3 | Translocation +; (20p13); (pp11-9p24::6p21.3-6qter) | H9 | 3, 1, 5 |  | p21.3 | Gain of 6p21.3 in skin samples from patients | MF | [2] |
|  | p21.3 | Segment der(22) | Mac2A | 5 |  |  |  |  |  |
|  | q10 | Segment (5pter-5p10::6q10-6q15) | PB2B | 5 |  |  |  |  |  |
|  | q10 | Segment (6qterq10, 6?6?, 2q22-23--2qter) | SeAx | x1(1), x2(4) |  |  |  |  |  |
|  | q13 | Segment (?p21q13) | H9/Hut78 | 1 |  | q13 | Translocation between unknown chromosome segment | SS | [4] |
|  |  |  |  |  |  | q13 | Translocation with 14p11 | SS | [4] |
|  |  |  |  |  |  |  |  |  |  |
|  | q15 | Segment (5pter-5p10::6q10-6q15) | PB2B | 5 |  | q15 | Loss of 5q15q22.1 (homozygous deletion) | MF | [1] |
|  | q15 | Deleted chromosome (-1)- terminal deletion with a break | Mac2A | 5 |  |  |  |  |  |
|  | q15 | Intersitial deletion with breakage and reunion (q23) | MyLa | 5 |  |  |  |  |  |
|  | q16-21 | Reciprocal translocation (11q21-22) B | Sez4/SZ4 | 5 |  | q16.3-21 | Loss of 6q16.3q21 | MF | [2] |
|  | q?21 | Uncertain paracentric inversion breakage and reunion (q?23) (6pterq?21, 6q?23q?21) | Hut102 | x1(3), x2(2) |  | q21 | Translocation with 8p23 | SS | [4] |
|  | q21 | Segment (2 copies) (6qter-6q21::10p11.1-10q24::6q21-6qter) | H9 | 3 |  | q21 | Deleted segment 6q21 | SS | [4] |
|  |  |  |  |  |  | q21 | Deleted segment 6q21 | MF | [4] |
|  |  |  |  |  |  | q21 | Deleted 6q21q25 |  | [3] |
|  |  |  |  |  |  |  |  |  |  |
|  | q22 | Translocation (6qter-6q22:2?2?:19q10-19qter) | SeAx | x1(2), x2(3) |  | q22 | Unbalanced translocation 17p10-116q22-23 | SS | [12] |
|  |  |  |  |  |  | q22.1 | Loss of 5q15q22.1 (homozygous deletion) | MF | [1] |
|  | q23 | Intersitial deletion with breakage and reunion (q23) | MyLa | 5 |  | q23 | Unbalanced translocation 17p10-116q22-23 | SS | [12] |
|  | q?23 | Uncertain paracentric inversion breakage and reunion (q?21) (6pterq?21, 6q?23q?21) | Hut102 | x1(3), x2(2) |  | q23 | Deletion of 6q23 in stage IVB patient | SS | [1] |
|  | ?q23 | Segment (2 copies) | H9/Hut78 | x1(1), x2(4) |  |  |  |  |  |
|  | q23-24 | Reciprocal translocation (14q23-24) B | HH | 5 |  | q24.2 | Loss of 6p24.1q24.2 | SS | [8] |
|  | q23-24 | Segment (6p21.1q23-24) | Sez4/SZ4 | 5 |  |  |  |  |  |
|  | q?27 | Uncertain paracentric inversion breakage and reunion (q?21) (6q?27q?21) | Hut102 | x1(3), x2(2) |  | q27 | Loss of 6q22.33q27 in stage IIB patient | MF | [1] |
|  |  |  |  |  |  | q27 | Addition of 6q27 in stage IVB patient | SS | [1] |
|  |  | Summary of structural aberrations seen in chromosome 6 across multiple cell lines: |  |  |  |  |  |  |  |
|  | 6?6?: Sez4/SZ4, SeAx, H9/Hut78, PB2B | |  |  |  |  |  |  |  |
|  | p10: Sez4, H9/Hut102 | |  |  |  |  |  |  |  |
|  | p21.1: Sez4/SZ4, Mac2A | |  |  |  |  |  |  |  |
|  | p21.3: Mac2A, H9 | |  |  |  |  |  |  |  |
|  | q23: MyLa, Hut102, H9/Hut78, HH, Sez4/SZ4 | |  |  |  |  |  |  |  |
|  | q15: PB2B, Mac2A, MyLa | |  |  |  |  |  |  |  |
|  |  |  |  |  |  |  |  |  |  |
| **7** | 7 (overall) | Trisomy | Sez4 | 5 | **7** |  |  |  |  |
|  | 7?7? | Segment (5pter-5p10:5q35-5q31:1?-1?:7?-7?:5q35-5q31) | Sez4/SZ4 | 5 |  |  |  |  |  |
|  | p11 | Translocation (19q13.4) | Sez4/SZ4 | 5, 4 |  | p11.1 | Gain of 7p11.1-q11.2 in skin sample from patient | MF | [2] |
|  | p11.2 | Paracentric inversion (7p22) | PB2B | 5 |  | p11.2 | Gain in 7p22p11.2 in 50% of patients | MF | [11] |
|  | p13 | Isochromosome for long arm | Sez4 | 5 |  | p13 | Gain in 7p13p14 in 45% of patients | MF | [10] |
|  | p13-15 | Translocation (14q10) | SZ4 | 5 |  | p14 | Loss of 7p14 | SS | [12] |
|  |  |  |  |  |  | p14 | Gain in 7p13p14 in 45% of patients | MF | [10] |
|  | p14-15 | Segment (10?) (2 copies); H9 (10q22.2-22.3, 2 copies) | Hut78/H9 | 5 |  | p14p15 | Gain in 7p14p15 in 41% of patients | MF | [10] |
|  |  |  |  |  |  | p14.3 | Gain of 7p14.3q22.1 | SS | [8] |
|  |  |  |  |  |  | p14.1 | Loss of 7p14.1 | SS | [12] |
|  | p15 | Deleted chromosome with break in band at 7p15 | MyLa | 5 |  |  |  |  |  |
|  | p22 | Paracentric inversion (7p11.2) | PB2B | 5 |  | p22 | Translocation with 9q13 | SS | [4] |
|  |  |  |  |  |  | p22 | Gain in 7p22p11.2 in 50% of patients | MF | [11] |
|  |  |  |  |  |  | p22 | Gain of 7p22 in stage IVB patient | SS | [1] |
|  |  |  |  |  |  | p22 | Gain in 7p21p22 in 45% of patients | MF | [10] |
|  | q11.2 | Translocation (8q24.1); (10q22.2-22.3)*H9(17? 2 copies) | Hut78/H9 | 5 |  | q11.2 | Gain in 7q11.2 in 50% of patients | MF | [10] |
|  |  |  |  |  |  | q11.2 | Gain of 7p11.1-q11.2 in skin sample from patients | MF | [2] |
|  | q22 | Translocation (4q23-27), SZ4 (20p11.1-11.2) | Sez4/Sz4 | x1(2), x2(2), 5 |  | q22 | Gain in 7q21q22 in 55% of patients | MF | [10] |
|  |  |  |  |  |  | p22.1 | Gain of 7p14.3q22.1 | SS | [8] |
|  | q31-32 | Segment (7pter-7q31-32:17?-17?) |  |  |  | q31 | Gain in 7q31 in 50% of patients | MF | [11] |
|  |  |  |  |  |  | q31 | Gain of 3q31 in 1 case | SS | [7] |
|  |  |  |  |  |  | q31 | Loss of 3q31 in 1 case | SS | [7] |
|  |  |  |  |  |  | q31 | Gain of 3q31 | MF | [7] |
|  | q32 | Segment (7pterq32, 10q22q24, 5p13-5pter) | SeAx | 5 |  | q32 | Gain in 7q32qter | SS | [9] |
|  |  |  |  |  |  | q32 | Translocation with unknown region/chromosome | MF/SS | [4] |
|  |  |  |  |  |  | q32 | Gain in 7q32q35 (55%) | MF | [10] |
|  |  | Summary of structural aberrations seen in chromosome 7 across multiple cell lines: |  |  |  |  |  |  |  |
|  | p15: SZ4, Hut78H9, MyLa | |  |  |  |  |  |  |  |
|  |  |  |  |  |  |  |  |  |  |
| **8** | 8 (overall) | Trisomy | HH | 5 | **8** | 8q | Gain in 8q-skin and lymph node samples from patients | MF | [2] |
|  | p21 | Translocation (11q23) * 2 copies | Sez4/SZ4 | 3 |  |  |  |  |  |
|  | p22 | Segment attached ?9?9 and 8p22qter | Mac2A | 5 |  |  |  |  |  |
|  | p22 | Translocation (?9?) | PB2B | 5 |  |  |  |  |  |
|  | p22-23 | Translocation with 5p13 (pterp22-23) | SeAx | 4 |  | p22 | Gain of 8p22pter in skin sample from patients | MF | [2] |
|  |  |  |  |  |  | p22 | Gain of 8p22 in 71% of patients | MF/SS | [7] |
|  |  |  |  |  |  | p23.3 | Gain in copy of 8p23.3-q24.3 | SS | [14] |
|  |  |  |  |  |  | p23 | Translocation with 6q21 | SS | [4] |
|  | p22-23 | Translocation with 10q24 (p22-23qter) *3 copies | SeAx | 5 |  | p23 | Translocation with 10p23 | SS | [4] |
|  | q10 | Segment with uncertain chromosome 5 (5?q33?qter) | Hut78 | 2 |  | q10 | Chromosomal gain 8q10qter in stage IIB and IVB patients | MF/SS | [6] |
|  |  |  |  |  |  | q10 | Inversion of 8q10 | SS | [1] |
|  | q11.2 | Translocation (18p11.3) *2copies | Sez4/SZ4 | 5 |  | q11 | Breakpoint at 8q11; translocation with 17p11 |  | [5] |
|  |  |  |  |  |  | q11 | Loss of 8q11 | SS | [7] |
|  | q?13 | Acrocentric (8?q13?q22) | Sez4/SZ4 | 5 |  | q11.2 | Gain in 8q11.2q12 in 60% of patients | SS | [12] |
|  | q22-24.1 | Segment with uncertain chromosome 5 (5?q33?qter) | Hut78 | 2 |  | q22 | Gain in 8q22q23 in 70% of patients | SS | [12] |
|  |  |  |  |  |  | q23 | Gain in 8q23q24.3 in 41% of patients | SS | [11] |
|  | q22 | Acrocentric (8?q13?q22) | Sez4/SZ4 | 5 |  | q24 | Gain of 8q24 | MF | [2] |
|  | q24.1 | Translocation with 7q11.2 | Hut78 | 5 |  | q24 | Translocation with 14q32.1 | MF | [4] |
|  |  |  |  |  |  | q24 | Translocation with 1p36 in stage IVB patient | SS | [1] |
|  |  |  |  |  |  | q24 | Amplification of 8q24 in stage IIB patient | MF | [1] |
|  |  |  |  |  |  | q24.1 | Gain in 8q24.1q24.3 in 75% of patients | SS | [12] |
|  | q24.1 | 2 copies of segment with chromosome 2 | Hut78 | 5 |  | q24.13 | Gain of 8q24.13q24.23 | SS | [8] |
|  | q24.1-24.2 | Translocation with uncertain chromosome 2; 2 copies of segment with chromosomes 2 | H9 | 3; 5 |  | q24.2 | Gain in copy number of 8q24.2 in 32% of patients | MF | [10] |
|  |  |  |  |  |  | q24.23 | Gain of 8q24.13q24.23 | SS | [8] |
|  |  |  |  |  |  | q24.3 | Gain in 8q23q24.3 in 41% of patients | SS | [11] |
|  |  |  |  |  |  | q24.3 | Gain of 8q24.3qter in skin samples | MF | [2] |
|  |  |  |  |  |  | q24.3 | Gain in copy of 8p23.3-q24.3 | SS | [14] |
|  |  |  |  |  |  | 24 (.1,.2,.3) | Gain in 8q24.1q24.3 in 75% of patients | SS | [12] |
|  |  | Summary of structural aberrations seen in chromosome 8 across multiple cell lines: |  |  |  |  |  |  |  |
|  | p22: Mac2A, PB2B, SeAx | |  |  |  |  |  |  |  |
|  | q22: Hut78, Sez4/SZ4 | |  |  |  |  |  |  |  |
|  | q24.1: Hut78/H9 | |  |  |  |  |  |  |  |
|  |  |  |  |  |  |  |  |  |  |
| **9** | ?9?9 | Uncertain segment (?9?-?9?:8p22-8qter) | PB2B | 5 | **9** |  |  |  |  |
|  | p10 | Translocation with uncertain short arm of an acrocentric chromosome (13, 14, 15, 21, 22) | |  |  | 9p | Loss of 9p | MF | [2] |
|  | p11 | Segment (9p24p11) (2 copies) | H9/Hut78 | 5 |  | p11.1 | Loss of 9p11.1p13 in 32% of patients | MF | [10] |
|  | p21 | Terminal deletion | MyLa | 5 |  | p21 | Loss of 9p21 in 41% of patients | MF | [10] |
|  |  |  |  |  |  | p21 | Loss of 9p21 in 30% of patients | MF | [11] |
|  |  |  |  |  |  | p21.1 | Loss of 9p21.3p21.2 in stage IIB patient | MF | [1] |
|  | p21-22 | Translocation with Y chromosome | H9/Hut78 | x1(2), x2(3) |  | p21.3 | Gain of 9p24.2p21.3 in stage IVB patient | SS | [1] |
|  |  |  |  |  |  | p21 | Homozygous deletion in 9p21.3p21.2 | SS | [14] |
|  |  |  |  |  |  | p21 | Loss of 9p21p22 | MF | [2] |
|  |  |  |  |  |  | p21 | Loss of 9p21 | MF | [2] |
|  |  |  |  |  |  | p21.3 | Loss of 9p21.3 in stage IVB patient | SS | [1] |
|  | p22 | Translocation (21q22) | SZ4/Sez4 | 5 |  | p22 | Loss of 9p21p22 | MF | [2] |
|  | p13-22 | Translocation (14q13) (2 copies) | SZ4/Sez4 | 5 |  |  |  |  |  |
|  | p24 | Segment (8pterp22, 9p24q34 and ?5?5) | Mac2A | 5 |  |  |  |  |  |
|  | p24 | Segment (9p24q22) | PB2B | 5 |  | p24.2 | Gain of 9p24.2p21.3 in stage IIB patient | MF | [1] |
|  | p24 | Segment (9p24p11) | H9/Hut78 | 5 |  |  |  |  |  |
|  | q12 | Deleted segment | Sez4 | 5 |  | q12 | Diminished 9q12q34 | SS | [3] |
|  |  |  |  |  |  |  |  |  |  |
|  | q13-21 | Segment | SeAx | 5 |  | q13 | Loss in 9q13-q21.33 in 32% of patients | SS | [14] |
|  |  |  |  |  |  | q13-q22 | Deletion of 9q13 q22 in stage IVB patient | SS | [1] |
|  |  |  |  |  |  | q13 | Translocation with 7p22 | MF/SS | [4] |
|  |  |  |  |  |  | q21 | Chromosomal gain 9q10q21 in stage IVB patient | SS | [6] |
|  |  |  |  |  |  | q21.31 | Loss 9q21.3q22.1 in stage IVA patient | SS | [1] |
|  |  |  |  |  |  | q21 | Loss of 9q21q22 in 35% of patients | MF | [11] |
|  |  |  |  |  |  | q21.1 | Loss of 9q21.1q31.3 in stage IVB patient | SS | [1] |
|  |  |  |  |  |  | q22 | Diminished 9q22q33 | SS | [3] |
|  | q22 | Translocation (21p12); Segment (9p24q22) | PB2B | 5 |  | q22 | Loss in 9q22q31 in 32% of patients | MF | [10] |
|  |  |  |  |  |  | q22 | Enhanced 9q22q33 | SS | [3] |
|  |  |  |  |  |  | q22 | Chromosomal loss in 9q22 in stage IVB patient | SS | [6] |
|  |  |  |  |  |  | q22.1 | Loss 9q21.3q22.1 in stage IVA patient | SS | [1] |
|  |  |  |  |  |  | q22 | Deletion of 9q13q22 in stage IVB patient | SS | [1] |
|  |  |  |  |  |  | q22 | Loss of 9q21q22 in 35% of patients | MF | [11] |
|  |  |  |  |  |  | q22.1 | Gain of 922.1q22.2 in stage IVA patient | SS | [1] |
|  | q34 | Segment (8pterp22, 9p24q34 and ?5?5) | Mac2A | 5 |  | q34 | Translocation with unknown chromosome segment | MF/SS | [4] |
|  |  |  |  |  |  | q34.11 | Gain of 9q34.11qter in stage IVA patient | MF/SS | [1] |
|  |  |  |  |  |  | q34.11 | Gain of 9q34.1qter in skin sample | MF | [2] |
|  |  |  |  |  |  | q34 | Loss of 9q34 | MF | [7] |
|  |  |  |  |  |  | q34 | Diminished 9q12q34 | SS | [3] |
|  |  |  |  |  |  | q34 | Loss of 9q34 in 1 case | SS | [7] |
|  |  |  |  |  |  | q34 | Gain of 9q34 in 1 case | SS | [7] |
|  |  |  |  |  |  | q34 | Chromosomal gain 9q33q34 in stage IVB patient | SS | [6] |
|  |  | Summary of structural aberrations seen in chromosome 9 across multiple cell lines: |  |  |  |  |  |  |  |
|  | p21: MyLa, H9/Hut78, SZ4/Sez4 | |  |  |  |  |  |  |  |
|  | p22: H9/Hut78, SZ4/Sez4, | |  |  |  |  |  |  |  |
|  | p24: Mac2A, PB2B, H9/Hut78 | |  |  |  |  |  |  |  |
|  |  |  |  |  |  |  |  |  |  |
| **10** | 10?10? | Translocation (12p12-13) | SeAx | 5 | **10** | 10p | Loss of 10p | MF | [2] |
|  | 10?10? | Segment (10?-10?:7p14-15-7qter) (2 copies) | Hut78/H9 | 5 |  |  |  |  |  |
|  | p11.2 | Segment (10q11.2q24) | H9 | 3 |  | p11 | Monosomy for region 10p11p15 |  | [5] |
|  |  |  |  |  |  | p11 | Loss of 10p11p13 | MF | [2] |
|  |  |  |  |  |  | p11.11 | Loss of 10p12,1p11.11 in stage IIB patient | MF | [1] |
|  |  |  |  |  |  | p11.2 | Loss of 10p11.2 | MF | [11] |
|  |  |  |  |  |  | p11.2 | Loss of 10p12p11.2 in 41% of patients | SS | [11] |
|  |  |  |  |  |  | p11.2 | Loss of 10p11.2 | SS | [12] |
|  |  |  |  |  |  | p11.2 | Loss of p11.2 in 41% of patients | SS | [11] |
|  |  |  |  |  |  | p11.21 | Loss of 10p11.21p13 in stage IVA patient | SS | [1] |
|  |  |  |  |  |  | p11.22 | Loss of 10p12.1p11.22 | SS | [8] |
|  |  |  |  |  |  | p14 | Diminished 10p13p14 | MF | [3] |
|  | p14 | Translocation (13q13) | MyLa | 5 |  | p14 | Gain 10p14p12.36 in stage IIB patient | MF | [1] |
|  | q10 | Translocation (6?) | PB2B | 5 |  | q10 | Loss 10q10qter in stage IVB | SS | [6] |
|  | q22 | Segment 10q22q24 | SeAx | 5 |  | q22 | Chromosomal loss 10q22qter in stage IVB patient | SS | [6] |
|  |  |  |  |  |  | q22 | Loss of 10q22q24 | MF | [2] |
|  |  |  |  |  |  | q22 | Deleted 10q22q26 | SS | [3] |
|  |  |  |  |  |  | q22.1 | Loss of 10q22.1 in stage IIB patient | MF | [1] |
|  | q22 |  |  |  |  | q22 | Loss of 10q2210q26 | SS | [12] |
|  | q22.2-22.3 | Translocation (7q11.2) (2copies) | Hut78/H9 | 5 |  | q22 | Loss of 10q22q24 in 47% of patients | SS | [11] |
|  | q24 | Translocation (10pter10q24) with (5q13) (2 copies) | SeAx | 5 |  | q24 | Translocation with 2p13 | SS | [4] |
|  | q24 | Translocation (10q2410qter) with (8p22-23) (3 copies) | SeAx | 5 |  | q24 | Loss of 10q22q24 in 47% of patients | SS | [11] |
|  | q24 | Segment (10pterq24)- 2 copies | Sez4/SZ4 | 5 |  |  |  |  |  |
|  | q24 | Translocation (4q28 (3); 4p15 (3)) | Sez4/SZ4 | # |  | q24 | Loss of 10q24q25 | SS | [12] |
|  | q24 | Translocation (10qter-10q24:15p12-15qter) | Sez4/SZ4 | 5 |  | q24.1 | Deletion of 10q23.33q24.1 |  | [15] |
|  | q24 | Segment (10qterq24); 4 copies in H9 | Hut78/H9 | x1(1), x2(4) |  | 24.32 | Loss of 10q24.32q26.3 | SS | [8] |
|  | q24 | Segment (10pterq24) | Hut78/H9 | 5; x2(4), x1(1) |  | q24.32 | Gain in copy of 10q24.32-q26.3 | SS | [14] |
|  | q24 | Break in band (10q24qter) Translocated to 9:5 | HH | 5 |  | q24.33q25.1 | Deletion of 10q24.33q25.1 |  | [15] |
|  | q25.3-26.1 | Reciprocal translocation (2p24) B | PB2B | 5 |  | q25 | Loss of 10q25q26 in 41% of patients | SS | [11] |
|  |  |  |  |  |  | q25 | Diminished 10q21q25 | SS | [3] |
|  |  |  |  |  |  | q25 | Loss of 10q25 | SS | [12] |
|  |  |  |  |  |  | q26 | Loss of 10q26 in 40% of patients | MF | [11] |
|  |  |  |  |  |  | q26 | Deleted 10q22q26 | SS | [3] |
|  |  |  |  |  |  | q26 | Loss of 10q2210q26 | SS | [12] |
|  |  |  |  |  |  | q26 | Loss of 10q26 in 15% of patients | SS | [3] |
|  |  |  |  |  |  | q26 | Loss of 10q25q26 in 41% of patients | SS | [11] |
|  |  |  |  |  |  | q26.3 | Loss of 10q23.31q26.2 in stage IIB patient | MF | [1] |
|  |  |  |  |  |  | q26.3 | Loss of 10q24.32q26.3 | SS | [8] |
|  |  | Summary of structural aberrations seen in chromosome 10 across multiple cell lines: |  |  |  |  |  |  |  |
|  | 10?10?: SeAx, Hut78/H9 | |  |  |  |  |  |  |  |
|  | q24: SeAx, Sez4/SZ4, Hut78/H9, HH | |  |  |  |  |  |  |  |
|  |  |  |  |  |  |  |  |  |  |
| **11** | ?11?11 | Uncertain segment with chromosomes 3 and 9 | SeAx | 5 | **11** |  |  |  |  |
|  | ?11?11 | Uncertain segment (19p13.1-13.2) | MyLa | 5 |  |  |  |  |  |
|  | ?11?11 | Uncertain segment (17q25) | HH | 5 |  |  |  |  |  |
|  | p10 | Uncertain segment (?16?16, 11?p12-14p10, 11q21qter) | Hut78 | 3 |  | p10 | Loss 11p10pter in stage IVB | SS | [6] |
|  | p11.2 | Segment with chromosome 4, 16 | Hut78 | 5 |  | p11.2 | - | MF/SS | [4] |
|  | ?p12-14 | Uncertain segment (?16?16, 11?p12-14p10, 11q21qter) | Hut78 | 3 |  |  |  |  |  |
|  | p15 | Segment (:14q?32-14q?24:11p15-11q21-22:) | SZ4/Sez4 | 5; x1(1),x2(3) |  | p15 | Translocation with unknown chromosome segment | MF | [4] |
|  |  |  |  |  |  | p15 | Gain of 11p15 | MF/SS | [7] |
|  | p15.2-15.5 | Deleted chromosome segment | PB2B | 4 |  | p15.1 | Gain of 11p15.1 | SS | [8] |
|  | q13-14 | Segment (11qter-11q13-14:21q10-21qter) | H9/Hut78 | 4 |  | q13 | Loss of 11q13 | MF/SS | [7] |
|  | q?13-14 | Uncertain terminal deletion with break (11pter-q?13-14) (2 copies) | H9 | 5 |  | q14.1 | Gain of 11q12-q14.1 in skin sample | MF | [2] |
|  | q14-21 | Translocation (2?) | SeAx | 5 |  | q14 | Gain 11q14qter in stage IIB | MF | [6] |
|  | q14-21 | Segment (11qter-11q14-21:2?-2?:2?p25-2?qter) | SeAx | 3 |  | q14 | Loss 11q14qter in stage IIB | MF | [6] |
|  | q21 | Uncertain segment (?16?16, 11?p12-14p10, 11q21qter) | Hut78 | 3 |  | q21 | Addition of 11q21 |  |  |
|  | q21-22 | Segment (:14q?32-14q?24:11p15-11q21-22:) | SZ4 | 5 |  |  |  |  |  |
|  | q21-22 | Reciprocal translocation (6q16-21) | SZ4/Sez4 | 5 |  |  |  |  |  |
|  | q22 | Translocation (14q13-21) | Mac2A | 5 |  | q22 | Loss of 11q22q23 (30%) | SS | [11] |
|  | q23 | Three-break reciprocal translocation (1q42, 8p21) *segment 11q23qter | SZ4/Sez4 | 5, 2 |  | q23 | Loss in 11q11q23 in stage IVA | SS | [6] |
|  |  |  |  |  |  | q23 | deletion of 11q23q25 | SS | [3] |
|  |  |  |  |  |  | q23 | Loss of 11q23 | SS | [7] |
|  |  |  |  |  |  | q23 | Loss of 11q22q23 (30%) | SS | [11] |
|  | q23 | Translocation (8p21) | Sez4 | 2 |  | q23.2 | Gain of 11q23.2qter in stage IVA patient | MF/SS | [1] |
|  |  | Summary of structural aberrations seen in chromosome 11 across multiple cell lines: |  |  |  |  |  |  |  |
|  | ?11?11: SeAx, MyLa, HH | |  |  |  |  |  |  |  |
|  | p15: SZ4/Sez4, PB2B | |  |  |  |  |  |  |  |
|  | q14: H9/Hut78, SeAx | |  |  |  |  |  |  |  |
|  | q21: SeAx, Hut78, SZ4/Sez4 | |  |  |  |  |  |  |  |
|  | q22: SZ4/Sez4, Mac2A | |  |  |  |  |  |  |  |
|  |  |  |  |  |  |  |  |  |  |
| **12** | 12 (overall) | Trisomy | Hut78/H9 | 1, 2 | **12** |  |  |  |  |
|  | 12 (overall) | Trisomy | Sez4/SZ4 | 5 |  |  |  |  |  |
|  | 12?12? | Uncertain translocation (18q22) | PB2B | 4 |  |  |  |  |  |
|  | p12-13 | Translocation (10? Or 14p12) | SeAx | 5, 4 |  | p12 | Deletion of 2p12 | MF | [4] |
|  |  |  |  |  |  | p12 | Gain of 12p12 | SS | [7] |
|  | p13 | Uncertain terminal deletion with break (12p13qter) | Hut78 | 2 |  | p13 | Translocation with 12p13, 21p11 and 19q13.3p13.1 | MF | [4] |
|  | q11 | Interstitial deletion with breakage and reunion (12pterq11 an 12q13qter) | Mac2A | 5 |  |  |  |  |  |
|  | q11 | Segment with break and reunion (12pterq13, 12q13q11, 12q13qter) | PB2B | 5, 4 |  |  |  |  |  |
|  | q13 | Interstitial deletion with breakage and reunion (12pterq11 an 12q13qter) | Mac2A | 5 |  | q13 | Gain of 12 q13 | SS | [7] |
|  | q13 | Segment with break and reunion (12pterq13, 12q13q11, 12q13qter) | PB2B | 5, 4 |  | q13 | Addition of 12q13 | SS | [3] |
|  | q21 | Translocation (3q11.2qter; 3pterp10) B (12pterq21; 12q21qter) | HH | 5, 4 |  | q21 | Deletion of 12q12q21 in 13/20 abnormal Chromosome 12 in clonal cells | MF | [13] |
|  | q21 | Segment (10pter-10q2?6:10q2?6-10q2?1:12q21-12qter) **GAIN OF SEGMENT | Mac2A | 5 |  | q21 | Translocation 12q21 with 18q21 in 100% abn. Chrom. 12 clonal cells | SS | [13] |
|  | q21-23 | Segment (12qter-12q21-23:1p21-1q21) **GAIN OF SEGMENT | SeAx | 5 |  | q22 | Loss of 12q13q22 | MF | [2] |
|  |  |  |  |  |  | q23 | Diminished 12q15q23 : 68% deleted in cells with NAV3 |  | [13] |
|  |  | Summary of structural aberrations seen in chromosome 12 across multiple cell lines: |  |  |  |  |  |  |  |
|  | Trisomy 12: Sez4/SZ4, H9/Hut78 | |  |  |  |  |  |  |  |
|  | p13: SeAx, Hut78 | |  |  |  |  |  |  |  |
|  | q11: Mac2A, PB2B | |  |  |  |  |  |  |  |
|  | q13: Mac2A, PB2B | |  |  |  |  |  |  |  |
|  | q21: HH, Mac2A, SeAx | |  |  |  |  |  |  |  |
|  |  |  |  |  |  |  |  |  |  |
| **13** | (-) 13 | Missing chromosome 13 | Hut78/H9 | # | **13** |  |  |  |  |
|  | (-) 13 | Missing chromosome 13 | SeAx | # |  |  |  |  |  |
|  | p12 | Segment (13q?31-13q?14:13p12-13qter) | Sez4/SZ4 | 3, 4 |  |  |  |  |  |
|  | q13 | Translocation of 13qterq13 (10p14) | MyLa | 5 |  | q13 | Loss of 13q13q21.2 | MF | [2] |
|  | q14 | Translocation of 13pterq14 (16?q23) | MyLa | 5 |  | q14 | Translocation with 1q44 | SS | [4] |
|  | q14 | Reciprocal translocation (Xp11.2-11.4) | Hut78 | x1(1), x2(4) |  | q14 | Deletion of 13q14q32 | SS | [3] |
|  | q14 | Translocation (Xp11.2-11.4) (2 copies) | H9 | 5 |  | q14 | Loss of 13q14q31 | MF | [2] |
|  | q14 | Segment q14qter | H9/Hut78 | 5 |  | q14 | Loss in 13q14q31 in 36% of patients | MF | [10] |
|  |  |  |  |  |  | q14 | Loss of 13q14 | SS | [12] |
|  |  |  |  |  |  | q14.2 | Homozygous deletion at 13q12.11q14.2 | SS | [14] |
|  | q?14 | Segment (13q?31-13q?14:13p12-13qter) | Sez4/SZ4 | 3, 4 |  | q14.3 | Loss of 13q12.11q14.3 | SS | [8] |
|  | q?31 | Segment (13q?31-13q?14:13p12-13qter); (17pter-17q25:3?q13.3-3q26:13q31-13qter) | Sez4/SZ4 | 3, 4; 3 |  | q31 | Loss in 13q14q31 in 36% of patients | MF | [10] |
|  |  |  |  |  |  | q31 | Diminished 13q31q34 | MF | [3] |
|  |  |  |  |  |  | q31 | Loss of 13q14.2q31 | MF | [2] |
|  | q33 | Translocation (14q21) | MyLa | 5 |  |  |  |  |  |
|  |  | Summary of structural aberrations seen in chromosome 13 across multiple cell lines: |  |  |  |  |  |  |  |
|  | q14: MyLa, H9/Hut78, SZ4/Sez4 | |  |  |  |  |  |  |  |
|  |  |  |  |  |  |  |  |  |  |
| **14** | p12 | Translocation (12p12-13) | SeAx | 5 | **14** |  |  |  |  |
|  | p12 | Segment 14p12q12-13 with chromosome 2 | MyLa | 5 |  |  |  |  |  |
|  | q10 | Short arm translocation to chromosome 9 (?acro-p) *14q13q10 | HH | 5 |  |  |  |  |  |
|  | q?11.2 | Segment from deletion (14q?11.2q?24) - 2 copies *11qterp11?11.2 | Hut 78/H9 | 5 |  |  |  |  |  |
|  | q12-13 | Translocation (2q32-33) | MyLa | 5 |  |  |  |  |  |
|  | q13 | Short arm translocation to chromosome 9 (?acro-p) *14q13q10 or 14q13qter | HH | 5 |  |  |  |  |  |
|  | q13 | Translocation 14q13qter with (9p13-22) - 2 copies | Sez4/SZ4 | 5 |  |  |  |  |  |
|  | q13-21 | Translocation (14q13-21qter) with (11q22) | Mac2A | 5 |  |  |  |  |  |
|  | q21 | Translocation of 14q21qter with (13q33) | MyLa | 5 |  |  |  |  |  |
|  | q22 | Translocation of 14pterq22 (18q22) | MyLa | 4 |  |  |  |  |  |
|  | q22 | Translocation of 14q22qter with (22q10) | PB2B | 5 |  |  |  |  |  |
|  | q?24 | Segment (:14q?32-14q?24:11p15-11q21-22) | Sez4/SZ4 | x1(1), x2(3); 5 |  |  |  |  |  |
|  | q?24 | Segment from deletion (14q?11.2q?24) - 2 copies *14q?24qter | Hut 78/H9 | 5 |  |  |  |  |  |
|  | q?32 | Segment (:14q?32-14q?24:11p15-11q21-22) | Sez4/SZ4 | x1(1), x2(3); 5 |  | q32 | Loss of 14q32 | MF/SS | [7] |
|  |  |  |  |  |  | q32.1 | Translocation with 8q24 | MF | [4] |
|  |  | Summary of structural aberrations seen in chromosome 14 across multiple cell lines: , |  |  |  |  |  |  |  |
|  | p12: SeAx. MyLa | |  |  |  |  |  |  |  |
|  | q13: MyLa, HH, Sez4/SZ4, Mac2A | |  |  |  |  |  |  |  |
|  | q21: Mac2A, MyLa | |  |  |  |  |  |  |  |
|  | q22: MyLa, PB2B | |  |  |  |  |  |  |  |
|  | q?24: Sez4/SZ4, Hut78/H9 | |  |  |  |  |  |  |  |
|  |  |  |  |  |  |  |  |  |  |
| **15** | 15 (overall) | Aneuploidy | HH | 5 | **15** |  |  |  |  |
|  | 15 (overall) | Trisomy | Hut78 | 2 |  |  |  |  |  |
|  | 15 (overall) | Tetraploid | Hut78 | 2 |  |  |  |  |  |
|  | 15?15? | Uncertain translocation (17p10) | SeAx | x1(1), x2(4) |  |  |  |  |  |
|  | p12 | Translocation (2p11.2) | Mac2A | 5 |  |  |  |  |  |
|  | p12 | Translocation (2p11.2) | PB2B | 5 |  |  |  |  |  |
|  | p12 | Segment (10qter-10q24::15p12-15qter) | SZ4/sez4 | 5 |  |  |  |  |  |
|  | q1?5 | Translocation (18q10) *15q1?5qter | H9 | 2 |  |  |  |  |  |
|  | q15 | Translocation (1q32; 1p36.2-36.3) | MyLa | 5 |  |  |  |  |  |
|  |  | Summary of structural aberrations seen in chromosome 15 across multiple cell lines: |  |  |  |  |  |  |  |
|  | p12: Mac2A, PB2B, SZ4/Sez4 | |  |  |  |  |  |  |  |
|  |  |  |  |  |  |  |  |  |  |
| **16** | 16?16? | Uncertain segment (6pter-6q22:2?-2?::6?-6?::16?-16?) | SeAx | 5 | **16** |  |  |  |  |
|  | p10 | Long arm of X and short arm of 16 fused at centromeres (Xq10) | SeAx | 5 |  |  |  |  |  |
|  | p10 | Pseudodicentric uncertain with segment 16pterp10 and 22p13qter | MyLa | 5 |  |  |  |  |  |
|  | p?11.1 | Uncertain segment with chromosome 4 and 13 | H9/Hut78 | 5 |  |  |  |  |  |
|  |  |  |  |  |  | p11.2 | Loss of 16p11.2 | SS | [12] |
|  | p?13.3 | Uncertain segment with chromosome 4 and 13 | H9/Hut78 | 5 |  | p13.2 | Gain 16p13.2p13.12 in stage IIB patient | MF | [1] |
|  |  |  |  |  |  | p13 | Translocation with 7q35 | MF | [4] |
|  | q11.1 | Terminal deletion | Sez4/SZ4 | 5, 3 |  |  |  |  |  |
|  | q11.2 | Interstitial deletion with break and reunion (16pterq11.2 and 16q22qter) | Mac2A | 5 |  |  |  |  |  |
|  | q11.2 | Interstitial deletion with break and reunion (16pterq11.2 and 16q22qter) | PB2B | 5 |  |  |  |  |  |
|  | q11.2-13 | Long arm of X and short arm of 16 fused at centromeres (6qter-6q22) | SeAx | 4 |  | q13 | Loss of 16q13q22 | MF | [2] |
|  | q21 | Segment (16pterq24 and 16q21qter) | Mac2A | 5 |  | q21 | Loss of 16q21q22 in 30% of patients | MF | [11] |
|  | ?q23 | Uncertain translocation (13q14) | MyLa | 5 |  | q23 | Loss of 16q23q24 in 35% of patients | MF | [11] |
|  |  |  |  |  |  | q23.1 | Loss of 16q23.1q24.3 in stage IVA patient | SS | [1] |
|  | q24 | Pseudodicentric uncertain with segment (16pter-16q24:1q22-25-1q10:1q10-1qter) | Sez4/SZ4 | 5 |  | q24 | Translocation with unknown chromosome segment | MF | [4] |
|  |  |  |  |  |  | q24.3 | Loss of 16q23.1q24.3 in stage IVA patient | SS | [1] |
|  |  |  |  |  |  | q24 | Diminished 16q22q24 | MF | [3] |
|  |  |  |  |  |  | q24 | Loss of 16q23q24 in 35% of patients | MF | [11] |
|  | q24 | Segment (16pterq24 and 16q21qter) | Mac2A | 5 |  | q24 | Translocation with 18q11 | MF | [4] |
|  |  | Summary of structural aberrations seen in chromosome 16 across multiple cell lines: |  |  |  |  |  |  |  |
|  | p10: SeAx, MyLa | |  |  |  |  |  |  |  |
|  | q11: Sez4/SZ4, Mac2A, PB2B, SeAx | |  |  |  |  |  |  |  |
|  | q24: Sez4/SZ4, Mac2A | |  |  |  |  |  |  |  |
|  |  |  |  |  |  |  |  |  |  |
| **17** | 17 (overall) | Tetraploid | Hut78/H9 | 5 | **17** | 17q | Gain of 17q in stage IIB patient | MF | [1] |
|  |  |  |  |  |  | 17q | Gain of 17q skin samples | MF | [2] |
|  | 17 (overall) | Trisomy | MyLa | 5 |  | 17p | Loss of 17p in stage IVA patient | SS | [1] |
|  |  |  |  |  |  | 17p | Loss of 17p | MF | [2] |
|  | 17 | Trisomy | Sez4 | 4 |  | 17q | Isochromosome of 17q corresponding to loss in 17p | MF | [2] |
|  | 17?17? | Segment (7pter-7q31-32::17?-17?) | H9 | 5 |  |  |  |  |  |
|  | 17?17? | Segment with chromosome 3, 11, 9 | SeAx | 5 |  |  |  |  |  |
|  | p10 | Translocation (15?) and (3p10) | SeAx | x1(1), x2(4) |  | p10 | Unbalanced translocation 17p10-116q22-23 | SS | [12] |
|  |  |  |  |  |  |  |  |  |  |
|  |  |  |  |  |  | p10 | Loss 17p10pter in stage IVB | SS | [6] |
|  | q10 | Translocation (3q12) | SeAx | 5 |  | q10 | Gain 17q10qter in stage IIB | MF | [6] |
|  | q10 | Isochromosome | SZ4/Sez4 | 3 |  |  |  |  |  |
|  | q10 | Segment (17qterq10, 17q10q25 and ?3?q13.3q21) | SZ4 | 3 |  |  |  |  |  |
|  | q25 | Segment (17pterq25, 3?q13.3q26 and 13q31qter) | SZ4 | # |  | q25 | Translocation with 1p32 | SS | [4] |
|  |  |  |  |  |  | q25 | Gain in 17q23q25 | SS | [12] |
|  |  |  |  |  |  | q25 | Enhanced 17q25 | SS | [3] |
|  | q25 | Translocation (?11q23) | HH | 5 |  | q25.3 | Gain of 17q11.2q25.3 | SS | [8] |
|  | q25 | Translocation (2p11.2), copy segment 17pterq25 | MJ | 5 |  | q25.3 | Gain copy of 17p11.2-q25 in 64% of patients | SS | [14] |
|  |  | Summary of structural aberrations seen in chromosome 17 across multiple cell lines: , |  |  |  |  |  |  |  |
|  | Trisomy: Sez4, MyLa | |  |  |  |  |  |  |  |
|  | 17?17?: SeAx, H9 | |  |  |  |  |  |  |  |
|  | q10: SeAx, Sez4/SZ4 | |  |  |  |  |  |  |  |
|  | q25: HH, MJ, SZ4 | |  |  |  |  |  |  |  |
|  |  |  |  |  |  |  |  |  |  |
| **18** | p10 | Translocation with another 18 chromosome (short arm of 2 #18 and long arm fused at centromeres) | H9 | 4 | **18** |  |  |  |  |
|  | p10 | Isochromosome for short arm 18 | Hut 78 | 4 |  |  |  |  |  |
|  | p11.3 | Translocation (8q11.2) -2 copies | Sez4/SZ4 | 5 |  | p11.3 | Loss 18p11.1p11.3 in stage IVA | SS | [6] |
|  | p11.3 | Translocation (6p12) | PB2B | 5 |  | p11.3 | Translocation of 18p11.3 with 12q24 | SS | [13] |
|  | q10 | Translocation (15q1?5) | H9 | 2 |  | q10 | Gain 18q10qter in stage IVA | SS | [6] |
|  | q10 | Inversion 18q10qter | H9 | 4 |  |  |  |  |  |
|  | q11.2 | Translocation 20?p11.2) | Hut78 | 2 |  | q11 | Translocation with 16q24 | MF | [4] |
|  | q11.2-11.3 | Translocation (2?p22-23) | Hut78 | x1(1), x2(4) |  |  |  |  |  |
|  | q12 | Translocation (8qter11.2, 18?p11.2q12) -2 copies | SZ4 | 5 |  |  |  |  |  |
|  | q22 | Translocation (14q22) *18q22qter | MyLa | 4 |  |  |  |  |  |
|  | q22 | Translocation (12?) *18pterq22 | PB2B | 5 |  |  |  |  |  |
|  | q22 | Translocation (3q24) *18pterq22 | HH | 5 |  |  |  |  |  |
|  | q23 | Translocation (?19p13.1-13.2) | MyLa | 5 |  |  |  |  |  |
|  |  |  |  |  |  |  |  |  |  |
|  |  | Summary of structural aberrations seen in chromosome 18 across multiple cell lines: |  |  |  |  |  |  |  |
|  | p11.3: Sez4/SZ4, PB2B | |  |  |  |  |  |  |  |
|  | q22: MyLa, PB2B, HH | |  |  |  |  |  |  |  |
|  |  |  |  |  |  |  |  |  |  |
| **19** | 19 (overall) | Trisomy | SeAx | 5 | **19** |  |  |  |  |
|  | p13.1-13.2 | Translocation (18q23) 19pter13.1-13.2 | MyLa | 5 |  | p13.2 | Loss 19p10p13.2 in stage IVA | MF/SS | [6] |
|  |  |  |  |  |  | p13 | Addition of 19p13 | SS | [3] |
|  |  |  |  |  |  | q13 | Gain of 19p13 | MF/SS | [7] |
|  |  |  |  |  |  | p13.2 | Gain of 19p13.2p11 in stage IIB patient | SS | [1] |
|  | p13.1-13.2 | Translocation (?11?) 19p13.1-13.2qter | MyLa | 4 |  | p13.1 | Translocation with 12p13, 21p11 and 19q13.3p13.1 | MF | [4] |
|  | q10 | Segment 19q10qter with 20q10- 2 copies | Hut78/H9 | 5 |  |  |  |  |  |
|  | q10 | Segment with chromosomes 2 and 6 | SeAx | x1(2), x2(3) |  |  |  |  |  |
|  | q13.?3 | Translocation (20q11.2) | Hut78/H9 | 5 |  |  |  |  |  |
|  | q13.4 | Translocation (7p11) 19pterq13.4 | SZ4/Sez4 | 4; x2(5) |  |  |  |  |  |
|  |  | Summary of structural aberrations seen in chromosome 19 across multiple cell lines: |  |  |  |  |  |  |  |
|  | q10: Hut78/H9, SeAx | |  |  |  |  |  |  |  |
|  |  |  |  |  |  |  |  |  |  |
| **20** | 20 (overall) | Tetraploid | SeAx | 5 | **20** |  |  |  |  |
|  | 20 (overall) | Tetraploid | Sez4/SZ4 | 5 |  |  |  |  |  |
|  | 20 (overall) | Trisomy | SZ4 | 3 |  |  |  |  |  |
|  | 20?20? | Uncertain segment (20pter-20q10::20?-20?-20q12-20qter) | Hut78/H9 | 2 |  |  |  |  |  |
|  | p10 | Segment (2p10) | Hut78 | 5 |  |  |  |  |  |
|  | p11.1-11.2 | Translocation (7q22) *20pterp11.1-11.2 | SZ4 | 3 |  | p11.22 | Loss in 20p13p11.22 in stage IIB patient | MF | [1] |
|  | ?p11.2 | Translocation (21q22.3) | MyLa | 5 |  |  |  |  |  |
|  | ?p11.2 | Translocation (18?q11.2) | Hut78 | 2 |  |  |  |  |  |
|  | p13 | Segment (20?q13.1?q13.1, 20p13q13.1 and 20q13.1qter) | Mac2A | 5 |  | p13 | Loss in 20p13p11.22 in stage IIB patient | MF | [1] |
|  | p13 | Segment (20?q13.1?q13.1, 20p13q13.1 and 20q13.1qter) | PB2B | 5 |  |  |  |  |  |
|  | p13 | Segment/ translocation (9p11-9p24::20p13-20qter) - 2 copies; (6p21.3) | Hut78/H9 | 5; x1(1), x2(3) |  |  |  |  |  |
|  | q10 | Segment with chromosome 8 and 5 | Hut78 | 2 |  | q10 | Loss 20q10qter in stage IVA/IVB | MF/SS | [6] |
|  | q10 | short arm of X and logn arm of 20 fused at centromeres | SZ4 | 2 |  | q10 | Gain 20q10qter in stage IVB | SS | [6] |
|  | q11.2 | Intersitial break and reunion (20q13.1) | Hut78/H9 | x1(1), x2(4) |  | q11.21 | Gain in 20q11.21q13.33 | SS | [8] |
|  | q13.1 | Intersitial break and reunion (20q13.1) | Hut78 | x1(1), x2(4) |  |  |  |  |  |
|  | q?13.1 | Translocation (4q33) *20?q13.1qter | SZ4 | 5 |  |  |  |  |  |
|  | q13.1 | Translocation with unknown chromosome segment | MyLa | 5 |  | q13 | Loss of 20q13 | SS | [7] |
|  | q13.1 | Segment (20?q13.1?q13.1, 20p13q13.1 and 20q13.1qter) | PB2B | 5 |  | q13 | Deletion of 20q11q13 | SS | [3] |
|  | q13.1 | Segment (20?q13.1?q13.1, 20p13q13.1 and 20q13.1qter) | Mac2A | 5 |  |  |  |  |  |
|  |  | Summary of structural aberrations seen in chromosome 20 across multiple cell lines: |  |  |  |  |  |  |  |
|  | Tetraploid: SeAx, Sez4/SZ4 | |  |  |  |  |  |  |  |
|  | p11.2: SZ4, MyLa, Hut78 | |  |  |  |  |  |  |  |
|  | p13: Mac2A, PB2B, Hut78/H9 | |  |  |  |  |  |  |  |
|  | q10: Hut78, SZ4 | |  |  |  |  |  |  |  |
|  | q13.1:Hut78, SZ4, MyLa, PB2B, Mac2A | |  |  |  |  |  |  |  |
|  |  |  |  |  |  |  |  |  |  |
| **21** | 21 (overall) | Trisomy | SeAx | 5 | **21** |  |  |  |  |
|  | p12 | Translocation (9q22) | PB2B | 5 |  |  |  |  |  |
|  | p12-13 | Pseudodicentric chromosomes with breaks and reunion (4q10) | SZ4 | 5 |  |  |  |  |  |
|  | q10 | Translocation (11q13-14) *q10-qter | H9/Hut78 | 4 |  |  |  |  |  |
|  |  |  |  |  |  | q22 | Addition of 21q22 | SS | [3] |
|  | q22 | Translocation q10-q22 with 11q13-14 | Hut78 | 4 |  | q22 | Gain of 21q22 | MF | [7] |
|  | q22 | Translocation (9p22) 2 copies (pterq22) | SZ4/Sez4 | 5 |  | q22.2 | Loss of 21q22.2qter | MF | [2] |
|  | q22.3 | Translocation (20?p11.2) | MyLa | 5 |  | q22.3 | Gain in 21q22.3 | SS | [8] |
|  |  | Summary of structural aberrations seen in chromosome 21 across multiple cell lines: |  |  |  |  |  |  |  |
|  | p12: PB2B, SZ4 | |  |  |  |  |  |  |  |
|  | q22: H9/ Hut78, SZ4/Sez4, MyLa | |  |  |  |  |  |  |  |
|  |  |  |  |  |  |  |  |  |  |
| **22** | 22 (overall) | Trisomy | Sez4/SZ4 | 5 | **22** |  |  |  |  |
|  | 22 (overall) | Trisomy | SeAx | 4 |  |  |  |  |  |
|  | p13 | Pseudodicentric chromosomes (116pterp10 and 22q13qter) | MyLa | 5 |  |  |  |  |  |
|  | q10 | Segment with attachment (6?pter?p21.3, 22q10q11.2 and 22q13.1qter) | Mac2A | 5 |  |  |  |  |  |
|  | q10 | Translocation (14q22) | PB2B | 5 |  |  |  |  |  |
|  | q10 | Whole arm translocation-long arm of 19 and 22 fused at centromeres (19q10) 2 copies | Hut78/H9 | 5 |  |  |  |  |  |
|  | q11.1 | Translocation (6p21.2-21.2) | Mac2A | 5 |  | q11 | Gain of 22q11 | MF/SS | [7] |
|  | q11.2 | Segment with attachment (6?pter?p21.3, 22q10q11.2 and 22q13.1qter) | Mac2A | 5 |  |  |  |  |  |
|  | q13.1 | Segment with attachment (6?pter?p21.3, 22q10q11.2 and 22q13.1qter) | Mac2A | 5 |  | q13 | Translocation with 3p21 in stage IVB patient | SS | [1] |
|  |  |  |  |  |  | q13 | Deletion of 22q12q13 | SS | [3] |
|  |  |  |  |  |  | q13 | Translocation with 3q25 | MF | [4] |
|  |  | Summary of structural aberrations seen in chromosome 22 across multiple cell lines: |  |  |  |  |  |  |  |
|  | q10: Mac2A, PB2B, Hut78/H9 | |  |  |  |  |  |  |  |
|  |  |  |  |  |  |  |  |  |  |
| **X** | X (overall) | Tetraploid | Hut102 | 5 | **X** |  |  |  |  |
|  | p10 | Segment from derivative (20q10) | SZ4 | 2 |  |  |  |  |  |
|  | p11.2-11.4 | Reciprocal translocation (13q14) | H9/Hut78 | 4 |  | p11.2 | Gain Xp11.2qter stage IIB | MF | [6] |
|  | p11.4-21 | Translocation (3q23-24) | SeAx | 4 |  | p11 | Loss of Xp11pter | MF | [2] |
|  | p22.2 | Reciprocal translocation (Xp22.3) | Sez4 | 2 |  |  |  |  |  |
|  | p22.3 | Reciprocal translocation (Xp22.2) | Sez4 | 2 |  |  |  |  |  |
|  | q10 | Whole arm translocaiton- long arm of X and short arm of 16 fused at centromeres (16p10) | SeAx | 5 |  |  |  |  |  |
|  | q23-24 | Segment (18, 8, 3) | SZ4 | 3 |  | Xq24 | Loss of Xq21q24 | MF | [2] |
|  |  | Summary of structural aberrations seen in chromosome X across multiple cell lines: |  |  |  |  |  |  |  |
|  | p11.4: H9/Hut78, SeAx | |  |  |  |  |  |  |  |
|  |  |  |  |  |  |  |  |  |  |
| **Y** | Y (overall) | Zero copies of Y (-) | SeAx | 5 | **Y** |  |  |  |  |
|  | Y (overall) | Zero copies of Y (-) | HH | 5 |  |  |  |  |  |
|  | Y (overall) | Zero copies of Y (-) | SZ4/Sez4 | 5 |  |  |  |  |  |
|  | Y (overall) | Zero copies of Y (-) | H9/Hut78 | 5 |  |  |  |  |  |
|  | Y (overall) | Zero copies of Y (-) | PB2B | 5 |  |  |  |  |  |
|  | Y (overall) | Zero copies of Y (-) | Mac2A | 5 |  |  |  |  |  |
|  | Y?Y? | Uncertain segment der(9))Y?-Y?::9p21-22-9qter) | H9/Hut78 | x1(2), x2(3) |  |  |  |  |  |
|  |  | Summary of structural aberrations seen in chromosome Y across multiple cell lines: |  |  |  |  |  |  |  |
|  | (-) Y (male cells): SeAx, HH, SZ4/Sez4, H9/Hut78, PB2B, Mac2A | |  |  |  |  |  |  |  |
|  |  |  |  |  |  |  |  |  |  |

**Supplementary Table 4.** Comparison of structural chromosomal aberrations between the studied CTCL cell lines and patient findings reported in literature based on 15 selected studies [1-15].

**References:**

1. Salgado R, Servitje O, Gallardo F, Vermeer MH, Ortiz-Romero PL, Karpova MB, Zipser MC, Muniesa C, Garcia-Muret MP, Estrach T, Salido M, Sanchez-Schmidt J, Herrera M, Romagosa V, Suela J, Ferreira BI, et al. Oligonucleotide array-CGH identifies genomic subgroups and prognostic markers for tumor stage mycosis fungoides. The Journal of investigative dermatology. 2010; 130(4):1126-1135.

2. Prochazkova M, Chevret E, Mainhaguiet G, Sobotka J, Vergier B, Belaud-Rotureau MA, Beylot-Barry M and Merlio JP. Common chromosomal abnormalities in mycosis fungoides transformation. Genes, chromosomes & cancer. 2007; 46(9):828-838.

3. Mao X, Lillington D, Scarisbrick JJ, Mitchell T, Czepulkowski B, Russell-Jones R, Young B and Whittaker SJ. Molecular cytogenetic analysis of cutaneous T-cell lymphomas: identification of common genetic alterations in Sezary syndrome and mycosis fungoides. The British journal of dermatology. 2002; 147(3):464-475.

4. Shapiro PE, Warburton D, Berger CL and Edelson RL. Clonal chromosomal abnormalities in cutaneous T-cell lymphoma. Cancer genetics and cytogenetics. 1987; 28(2):267-276.

5. Thangavelu M, Finn WG, Yelavarthi KK, Roenigk HH, Jr., Samuelson E, Peterson L, Kuzel TM and Rosen ST. Recurring structural chromosome abnormalities in peripheral blood lymphocytes of patients with mycosis fungoides/Sezary syndrome. Blood. 1997; 89(9):3371-3377.

6. Fischer TC, Gellrich S, Muche JM, Sherev T, Audring H, Neitzel H, Walden P, Sterry W and Tonnies H. Genomic aberrations and survival in cutaneous T cell lymphomas. The Journal of investigative dermatology. 2004; 122(3):579-586.

7. Mao X, Lillington DM, Czepulkowski B, Russell-Jones R, Young BD and Whittaker S. Molecular cytogenetic characterization of Sezary syndrome. Genes, chromosomes & cancer. 2003; 36(3):250-260.

8. Mao X and McElwaine S. Functional copy number changes in Sezary syndrome: toward an integrated molecular cytogenetic map III. Cancer genetics and cytogenetics. 2008; 185(2):86-94.

9. Barba G, Matteucci C, Girolomoni G, Brandimarte L, Varasano E, Martelli MF and Mecucci C. Comparative genomic hybridization identifies 17q11.2 approximately q12 duplication as an early event in cutaneous T-cell lymphomas. Cancer genetics and cytogenetics. 2008; 184(1):48-51.

10. van Doorn R, van Kester MS, Dijkman R, Vermeer MH, Mulder AA, Szuhai K, Knijnenburg J, Boer JM, Willemze R and Tensen CP. Oncogenomic analysis of mycosis fungoides reveals major differences with Sezary syndrome. Blood. 2009; 113(1):127-136.

11. Laharanne E, Oumouhou N, Bonnet F, Carlotti M, Gentil C, Chevret E, Jouary T, Longy M, Vergier B, Beylot-Barry M and Merlio JP. Genome-wide analysis of cutaneous T-cell lymphomas identifies three clinically relevant classes. The Journal of investigative dermatology. 2010; 130(6):1707-1718.

12. Vermeer MH, van Doorn R, Dijkman R, Mao X, Whittaker S, van Voorst Vader PC, Gerritsen MJ, Geerts ML, Gellrich S, Soderberg O, Leuchowius KJ, Landegren U, Out-Luiting JJ, Knijnenburg J, Ijszenga M, Szuhai K, et al. Novel and highly recurrent chromosomal alterations in Sezary syndrome. Cancer research. 2008; 68(8):2689-2698.

13. Karenko L, Sarna S, Kahkonen M and Ranki A. Chromosomal abnormalities in relation to clinical disease in patients with cutaneous T-cell lymphoma: a 5-year follow-up study. The British journal of dermatology. 2003; 148(1):55-64.

14. Caprini E, Cristofoletti C, Arcelli D, Fadda P, Citterich MH, Sampogna F, Magrelli A, Censi F, Torreri P, Frontani M, Scala E, Picchio MC, Temperani P, Monopoli A, Lombardo GA, Taruscio D, et al. Identification of key regions and genes important in the pathogenesis of sezary syndrome by combining genomic and expression microarrays. Cancer research. 2009; 69(21):8438-8446.

15. Wain EM, Mitchell TJ, Russell-Jones R and Whittaker SJ. Fine mapping of chromosome 10q deletions in mycosis fungoides and sezary syndrome: identification of two discrete regions of deletion at 10q23.33-24.1 and 10q24.33-25.1. Genes, chromosomes & cancer. 2005; 42(2):184-192.
