# Supplementary material for: Analysis of CTCL cell lines reveals important differences between mycosis fungoides/Sézary syndrome vs. HTLV-1+ leukemic cell lines
Source: Oncotarget. 2017 Oct 7;8(56):95981–98. doi: 10.18632/oncotarget.21619 (PMC5707075; doi:10.18632/oncotarget.21619)
Supplement: Supplementary file 6 [file oncotarget-08-95981-s006.docx]

| **Gene Name** | **Forward Primer** | **Reverse Primer** |
| --- | --- | --- |
| ACTB | CTGGAACGGTGAAGGTGACA | AAGGGACTTCCTGTAACAATGCA |
| AHI1 | GTCCAAAACTACCCCATCAAGGCT | GCAGCACAGGAACGTATCACCT |
| BCL7A | GAACCATGTCGGGCAGGTCG | CCCATTTGTAGATTCGTAGGGATGTGT |
| BIRC5 | TTCTCAAGGACCACCGCATCT | AGTGGATGAAGCCAGCCTCG |
| CCL18 | CCCTCCTTGTCCTCGTCTGCA | GCACTGGGGGCTGGTTTCAG |
| CCL26 | TTCCAATACAGCCACAAGCCCC | GGATGGGTACAGACTTTCTTGCCTC |
| CCND2 | TCAAGTGCGTGCAGAAGGACAT | CTTCGCACTTCTGTTCCTCACA |
| CCR3 | ACGCTGCTCTGCTTCCTGG | TCCTCAGTTCCCCACCATCGC |
| CCR4 | AGCATCGTGCTTCCTGAGCAA | GGTGTCTGCTATATCCGTGGGGT |
| CD22 | CATCCTCATCCTGGCAATCT | CTCTGCATCTCCAGTTCGTG |
| CDKN1C | AGATCAGCGCCTGAGAAGTCGT | CTCGGGGCTCTTTGGGCTCT |
| CDO1 | ACAGTCCACCTTTTGATACATGCCA | GCCCCTTAGTTGTTCTCCAGCG |
| CHD1 | AGACCGACATCAGGGAGATTCTTACA | CCTGTGATCATCCAGTTTTCTGTGTTTC |
| CHD7 | GGCACAGCTCCACCCATCAC | CTGAGTCATATCCGGCACTGGTTT |
| CLU | CGCCACAACTCCACGGGCTG | GTCAACCTCTCAGCGACCTGGA |
| CNOT3 | CGTCCGTCTCCAAGAGAGTATGAAGA | CAAACTGCTCCACGCCCTCG |
| CST6 | CTGACGATGGAGATGGGGAGCA | GCCAGGGAACCACAAGGACC |
| CTAGE1 | TCCTTACCGTCCCCCAAGACCT | GCTGTCGTTCTGGATGTTCAGCA |
| DMAP1 | GCGCGGATGTACGGGACATTC | CCTCGGGCCTCTTGAAAGTCAG |
| E2F4 | AGATACCCTCTTGGCCATCCG | GTGAATCTGGTACTTCTTCTGCCC |
| EED | TGCGGCCAAGAAGCAGAAGC | TGCATTTGGCGTGTTTGTAGGTG |
| ENV | TCGACGCTCCAGGATATGAC | GAGGCTGGCACGATCGATAC |
| EP400 | TGCCCCCACCAAACCACAGA | TGCTTTCCTCAGCTCCGCAATG |
| EPHA4 | GGCAGATGGTGAATGGCTGGT | GAGTAGCTGTGGGGTGGGCA |
| ESRRB | CGGGGACATTGCCTCTGGCTA | TGATCTCGCACTCGTTGGTGGC |
| EZH2 | AAAATTATGATGGGAAAGTACACGGGGA | CTTCTCTTTCTTCAGGATCGTCTCCATC |
| FLT4 | CCTGACACGCTCTTGGTCAACA | CCGGTCATCCCACACCACCT |
| FOSL1 | CGGAGGAAGGAACTGACCGACT | TTCCAGCACCAGCTCTAGGCG |
| FOXP3 | AGCTCCTACCCACTGCTGGC | TGCCCTGCCCTTCTCATCCAG |
| FYB | CCTCCCTTGTTTACCTTGGGTCC | GTGGAGGTGGTGGCAGGGAA |
| GAG | TATGCAGACCATCCGGCTTG | TTGTTGGCTTGGACACGGAG |
| GNLY | TGGTCTTCTCTCGTCTGAGCCC | CCCAGCTCCTGTGTTTTGGTCA |
| GTSF1 | GCAGACCAGCACCCCATTTGTC | GGCAGAGATTTGGGAACTCGCA |
| IFNG | GCATCGTTTTGGGTTCTCTTGGC | CCGCTACATCTGAATGACCTGCA |
| IL1F7 | GGACAAAGTCATCCATCCCTTCAGC | CCGACTCCAGCATGTTCCAGG |
| IL2RA | GAAAGACCTCCGCTTCACTGCC | GGATCTCTGGCGGGTCATCGT |
| IL4 | GCAGTTCCACAGGCACAAGCA | GGTTGGCTTCCTTCACAGGACA |
| IL5 | GCTGATAGCCAATGAGACTCTGAGG | TCCACAGTACCCCCTTGCACA |
| IL9 | TGACCAGTTGTCTCTGTTTGGGC | TGGGTATCTTGTTTGCATGGTGGT |
| IL10 | AGGAGGTGATGCCCCAAGCTG | GCCTTGCTCTTGTTTTCACAGGG |
| IL12A | TGGCAGTTATTGATGAGCTGATGCA | AGCATGAAGAAGTATGCAGAGCTTGA |
| IL13 | GCATGGTATGGAGCATCAACCTGA | CCTCTGGGTCTTCTCGATGGCA |
| IL17A | GTCAACCTGAACATCCATAACCGGA | GCACTTTGCCTCCCAGATCACA |
| IL17F | TCACGTAACATCGAGAGCCGC | TGGAGATGTCTTCCTTTCCTTGAGCA |
| IL18 | TCATTGACCAAGGAAATCGGCCTC | TCACACTTCACAGAGATAGTTACAGCCA |
| IL21 | TCTGCCAGCTCCAGAAGATGTAGA | TCTCCCTGCATTTGTGGAAGGTG |
| IL21R | AAGGAAGGCTGGAACCCTCACC | GGGGCATGAAGAACCGCTCAG |
| IL22 | CCCTTGAAGAAGTGCTGTTCCCT | TCAGCTTTTGCACATTCCTCTGGA |
| IL26 | TCCTGTGCTTCATCAGCTAGAGAGA | GGCTTTGGTTTACTGACTGCTTTCC |
| IRF3 | CCAGCCAGACACCTCTCCGG | GCAGGGCTCAGGGGCTACAG |
| IRF4 | TATGCTTGTGCCCCACCTGAGT | ACGTGGTCAGCTCCTTCACGA |
| ITK | GGCTCAACAAGGACAAGGTGGC | TCCAGGCACACCCCATACAGC |
| JARID2 | AGGCTAGTGGAAGAGAAGGACTGC | CCTGTGTTATTGGGGAGGACGG |
| JUNB | GAACGCCTGATTGTCCCCAACA | CGAAGCCCTCCTGCTCCTCG |
| KAT5 | TCCTGAGCGTGAAGGACATCAGT | GCCTCTTTCTTGGGGAACTGGATC |
| KIT | GCACCGAAGGAGGCACTTACAC | GCTGCCACACATTGGAGCATG |
| KLF4_ | ATCTCAAGGCACACCTGCGAA | ATCTGAGCGGGCGAATTTCCAT |
| LCE2B | TGCTCCTGCGTGTGACCAGG | GGGGCAGGCATTTAGGGGGAC |
| LCK | GGAGATCTGGGCTTTGAGAAGGGG | GCCACAAAATTGAAGGGGATGAAGC |
| LEF1 | AGCGAATGTCGTTGCTGAGTGT | AGCTGTCTTTCTTTCCGTGCTAATTCA |
| LOR | CTCTCCTCACTCACCCTTCCTGG | CCACCGCCGCCAGAGGTCTT |
| LTBP4 | CGGCATCTGTACCAACACCGAC | CTGCGACCCGCACAGGG |
| MAX | ACGGGCTCATCATAATGCACTGG | TGTGGCTTTGTCTAGGATTTGGGC |
| MMP12 | GCCGTAATGTTCCCCACCTACAA | TCAGGATTTGGCAAGCGTTGGT |
| MPZL2 | TGGGTTTCCCTCATGTATGGCAAG | CATTAACAGCCTCCAGCACCCG |
| MTF2 | ACTGAGGGAACTGCACATTCATCC | GGCCAAGATCTTCCTGTACGCG |
| MXI1 | CCCGGCACACAACACTTGGTTT | CGCCACTTTAAAAATCTCTGTTCTCGTT |
| NAIP | TTCCTGGGTCCAGAGAGAATTACC | TCCAGCCGTAGTTCTTCGTAAGC |
| NANOG | TGGATCCAGCTTGTCCCCAAA | AGGCCCACAAATCACAGGCATA |
| NFKB1 | TGCAACTATGTGGGACCAGCAA | AGTGTTTTCCCACCAGGCTGT |
| NOTCH1 | AGCTGGACCCCATGGACG | GGTGGCACTCTGGAAGCACT |
| NR0B1 | AGGGGACCGTGCTCTTTAACCC | AGTTCGATGAATCTGTCATGGGGC |
| PHC1 | CCAAACACCAGCACTACACAGCA | GCACAGATTGGGTCAAGGTGGT |
| PLK1 | AGTACCTGCACCGAAACCGAGT | GGGTCTTCTTCCTCTCCCCGTC |
| PLS3 | ACTCTCTTGGTGTCAATCCTCACGT | TCCCAGTTTCGGGTATGGAGGT |
| POL | AGGCTTTGCAACACTTGGTC | GGATGAATCGCCAGGTTCCA |
| POU5F1 | TGCAAAGCAGAAACCCTCGT | TCGGGCACTGCAGGAACAAAT |
| PSORS1C2 | CAGCTTTGGGGGCCAGTACAT | CCTCTGCGGGTGGGTGAGAG |
| PX | GGCTCCGTTGTCTGCATGTA | AATCATAGGCGTGCCATCGG |
| REC8 | TGATGGAGACCCTAGAAGATGCTCC | ACTCTCTCTGGGATTGCAGCCT |
| RNF2 | GCCTCATCCCACACTTATGGAAAAAGA | AGTTCTTCTAAAGCTAACCTCACAGCC |
| SALL4 | ACCCCAGCATCTGGCTAAAACAC | GTGGCTTCATCCTCACTCGCCA |
| SDHA | TGGGAACAAGAGGGCATCTG | CCACCACTGCATCAAATTCATG |
| SELL | CAGGCAAATGGAACGATGACGC | ACCCCACATCACAGTTGCAGGT |
| SERPINB4 | ACCAGTGTGGAATCTACTGATTTTGCA | TCGTATCATTGCCAATAGTCCCATCAG |
| SERPINB13 | TGTGCTTCTGCCCAACGACATC | ACCGTCCTCCACCTCAAACCG |
| SH2D1A | AGTCCTCAGCTAGAAGTACACAAGGT | TGCATTTGTAGCTCACCGAACTGT |
| SOX2 | TGAACCAGCGCATGGACAGTTA | CATCATGCTGTAGCTGCCGTT |
| SPO11 | ACAGAGCAACACTTATGCAACCAAAAG | ACTCCTCCTTGACACTTTTAACATGCA |
| STAT1 | TGATCTCCAACGTCAGCCAGC | GCCAACTCAGCACTTCTGAAAGC |
| STAT2 | CATTGGAGGGCGCGGGGACT | TCGAATGTCCACAGGCAGGAGG |
| STAT3 | ATGCGGCCAGCAAAGAATCA | AGCGGCTATACTGCTGGTCAAT |
| STAT4 | GGAAATTCGGCATCTGTTGGCC | TTCTCTTTGGAAACACGACCTAACTGT |
| STAT5A | TGGCAGTGGTTTGACGGGGT | GTCGGGCTTGTTGATGAGCAGG |
| STAT5B | ACTGAAGATCAAGCTGGGGCA | ACAATATATGGCGGATGCAGCG |
| STAT6 | GGCCACTTTCAGACAAATACTTCAAGGA | TGCAGCCTCCGCAAGCCT |
| SUZ12 | TCATCGCCAACCTGGATTTGCT | ATGTTCTTTGCTGTTCTACTTCCCCAT |
| SYCP1 | CCACCAGCTTCTCATCTTTGTGTCA | AGCAATTACAGCCCAACGGTCC |
| TAX | GGTTGAGTGGAACGGAAGGA | GGTTGAGTGGAACGGAAGGA |
| TBX3 | CTGGAGGCTAAAGAACTTTGGGATCA | ATCCAGCCCAGAACATCTCACTTTAAAT |
| TBX21 | ACGCTTCCAACACGCATATCTTTACT | GTTCTCCCGGAATCCTTTGGCA |
| TCF3 | CCTGTTTGAAACGGCGAGAAGA | TGGGGAGCTGAAAGCACCAT |
| TCF7L1 | ACCGTATTACCCACTCTCTCCCG | ATCGAGGCGTTCATGGCGAG |
| TGFB1 | AGTTGTGCGGCAGTGGTTGA | CTTGCAGTGTGTTATCCCTGCT |
| THAP11 | CCAAAGGACGCTGAGTTGCGG | CGTACCGTGTAGGTCTTGCGG |
| TNFRSF8 | AGCTCCACCTGTGCTACCCG | CGTTGAGCTCCTCCTGGGTCTG |
| TOX | TGAGCATGACAGAGCCGAGCC | CAGCGAGTGGTCTGGGAGGG |
| TRAF3IP3 | TGACCACCTCTCCTCACAGGCT | TTGGTTTGCTGACTGGCATCGT |
| TRIM28 | CCCCACAGGAGTTTGCCCAG | GCACAGCAGAGAACTTGGTGTCA |
| TRRAP | GTCCACGCTGATGTTGGAGCA | AGGGAGTAAAGCTCCGCAAGGG |
| VEGFC | GCCAACCTCAACTCAAGGACAGA | CCCCACATCTATACACACCTCCCG |
| WIF1 | GAATTCCTGTCCTTGCGCTCCC | CTGCCACCCCATCCTGTTTTCC |
| XTP6 | AGAGTGGAGGCTGGAAGGATGG | TCAGCACAAGGCAAGGATGCTC |
| YWHAZ | TCCCCAATGCTTCACAAGCAGA | TCTTGTCATCACCAGCGGCAA |
| ZFX | GTTGAACTGCTTGATCAGAACAGCAG | TCGGCATGAAGGTTTTGATTTCATTGTC |

**Supplementary Table 5.** Primers used for RT-PCR experiments.
